# TRF2 and VEGF-A: an unknown relationship with prognostic impact on survival of colorectal cancer patients

**DOI:** 10.1186/s13046-020-01612-z

**Published:** 2020-06-15

**Authors:** Roberto Dinami, Manuela Porru, Carla Azzurra Amoreo, Isabella Sperduti, Marcella Mottolese, Simonetta Buglioni, Daniele Marinelli, Marcello Maugeri-Saccà, Andrea Sacconi, Giovanni Blandino, Carlo Leonetti, Giuliana Di Rocco, Alessandra Verdina, Francesca Spinella, Francesco Fiorentino, Gennaro Ciliberto, Annamaria Biroccio, Pasquale Zizza

**Affiliations:** 1 Oncogenomic and Epigenetic Unit, IRCCS – Regina Elena National Cancer Institute, Via Elio Chianesi 53, 00144 Rome, Italy; 2 Pathology Unit, IRCCS – Regina Elena National Cancer Institute, Rome, Italy; 3 Department of Biostatistics, IRCCS – Regina Elena National Cancer Institute, Rome, Italy; 4 Division of Medical Oncology 2, IRCCS - Regina Elena National Cancer Institute, Rome, Italy; 5 Division of Medical and Molecular Medicine, Sapienza - Università di Roma, Azienda Ospedaliera Sant’Andrea, Rome, Italy; 6 SAFU, IRCCS - Regina Elena National Cancer Institute, Rome, Italy; 7 Unit of Cellular Networks and Molecular Therapeutic Targets, IRCCS - Regina Elena National Cancer Institute, Rome, Italy; 8 GENOMA-Molecular Genetics Laboratory, Rome, Italy; 9 Scientific Direction, IRCCS - Regina Elena National Cancer Institute, Rome, Italy

**Keywords:** Colorectal cancer, TRF2, VEGF-A, Prognostic markers

## Abstract

**Background:**

Colorectal cancer is one of most common tumors in developed countries and, despite improvements in treatment and diagnosis, mortality rate of patients remains high, evidencing the urgent need of novel biomarkers to properly identify colorectal cancer high-risk patients that would benefit of specific treatments. Recent works have demonstrated that the telomeric protein TRF2 is over-expressed in colorectal cancer and it promotes tumor formation and progression through extra-telomeric functions. Moreover, we and other groups evidenced, both in vitro on established cell lines and in vivo on tumor bearing mice, that TRF2 regulates the vascularization mediated by VEGF-A. In the present paper, our data evidence a tight correlation between TRF2 and VEGF-A with prognostic relevance in colorectal cancer patients.

**Methods:**

For this study we sampled 185 colorectal cancer patients surgically treated and diagnosed at the Regina Elena National Cancer Institute of Rome and investigated the association between the survival outcome and the levels of VEGF-A and TRF2.

**Results:**

Tissue microarray immunohistochemical analyses revealed that TRF2 positively correlates with VEGF-A expression in our cohort of patients. Moreover, analysis of patients’ survival, confirmed in a larger dataset of patients from TCGA, demonstrated that co-expression of TRF2 and VEGF-A correlate with a poor clinical outcome in stage I-III colorectal cancer patients, regardless the mutational state of driver oncogenes.

**Conclusions:**

Our results permitted to identify the positive correlation between high levels of TRF2 and VEGF-A as a novel prognostic biomarker for identifying the subset of high-risk colorectal cancer patients that could benefit of specific therapeutic regimens.

## Background

Colorectal cancer (CRC) is considered a very important public health issue since it is the third most common cancer type diagnosed in men and the second most commonly occurring tumor in women [1]. Despite a substantial improvement of diagnosis and treatment, it still represents a major cause of tumor-related deaths worldwide [2]. Notably, the most recent epidemiological studies evidenced a gradual and continuous increase of this malignancy over the past years with a global incidence that, according to the World Health Organization GLOBOCAN database, in the 2018 exceeded 1.8 million of new cases, a trend that is predicted to still grow in the future [3].

Treatment of CRC patients (stage III, IV and high risk stage II) commonly consists in the surgical resection of the tumor and fluoropyrimidine-based chemotherapy (e.g. 5-fluorouracil (5-FU) or capecitabine) administered alone or in combination with oxaliplatin (FOLFOX), Irinotecan (FOLFIRI) or both (FOLFOXIRI) [4]. Moreover, addition of target therapy based on the administration of antibodies against the vascular endothelial growth factor (VEGF) or the epidermal growth factor receptor (EGFR), has demonstrated to further improve the clinical outcome of metastatic CRC patients [5]. Unfortunately, a certain number of CRC patients has demonstrated to not benefit of these therapeutic regimens [6].

Prediction of clinical outcome of CRC patients is mainly based on the evaluation of tumor stage, lymph-node positivity and presence of distant metastases [7]. Although these clinical criteria provide valuable prognostic information and guide therapy decisions, the response and outcome of individual patients is not fully predictable. This problem is particularly relevant for certain patients (particularly stage II and III) that, independently of their clinic-pathological characteristics, show a quite variable clinical course, indicating the urgent need of identifying novel biomarkers with clinical relevance [8–10]. In the last few years, single genetic characteristics – such as the mutational state of driver oncogenes (e.g. KRAS, NRAS, BRAF) – and molecular signatures based on somatic mutational profiling, were proposed as prognostic criteria to detect patients at a high risk of recurrence [11–13]. Moreover, genetic events, gene-expression profile and the tumor microenvironment were integrated to enable four consensus molecular subtypes [14]. Despite the huge efforts done for developing novel and effective prognostic criteria, these molecular markers are difficult to integrate with the current staging system.

Recently, telomere length has been accounted as a putative prognostic marker for solid tumors, included CRC [15, 16]. Telomeres are specialized nucleoprotein structures, located at the terminal portion of chromosomes, playing a central role in the maintenance of genomic integrity [17]. In humans, telomeres are composed of TTAGGG tandem repeats of DNA associated with a protein complex – Shelterin – constituted by six subunits (TRF1, TRF2, RAP1, TIN2, TTP1 and POT1) participating in telomere protection and inhibition of aberrant DNA damage response (DDR) [18]. Due to the linear nature of human chromosomes, telomeres undergo to progressive shortening during each cycle of cell division. Finally, when telomeres reach a critical length (Hayflick limit), they are no longer protected by the Shelterin complex and cells enter into a state of replicative senescence that, under normal conditions, can leads to cell death [19]. On the contrary, when protective mechanisms driven by tumor suppressor genes (e.g. TP53) are dysregulated, cells continue to proliferate by inducing chromosomal instability [20]. Since telomere erosion has been found accelerated in response to specific alterations of genes participating in the carcinogenesis of CRC (e.g. APC and MSH2), a marked telomere shortening has been considered an early event of CRC carcinogenesis [21]. Despite the reported observations would support the idea of a direct implication of telomere length in CRC, lack of solid evidences and a limited amount of available studies make its prognostic relevance object of an extensive debate [21].

In contrast to telomere length, prognostic value of Shelterin proteins has not extensively evaluated, so far. Our laboratory is long-lasting involved in the study of the telomeric proteins, with a particular interest for Telomere Repeat binding Factor 2 (TRF2). Besides its role in telomere maintenance, TRF2 has been recently found to localize also outside telomeric regions, where it can affect the expression of multiple target genes [22–24]. TRF2 is regulated by the Wnt/β-catenin pathway [25], is relevant in oncogenesis of CRC [26–28], and is over-expressed in several human malignancies [29–32], included CRC, in which levels of TRF2 have been found to increase during the progression from normal mucosa to focal adenocarcinomas [24]. Moreover, recent experimental data from our and other laboratories have evidenced a tight correlation between TRF2 and the vascularization mediated by VEGF-A [24, 33, 34]. VEGF-A – one of the main mediators of angiogenic response – is not validated, per se, as a prognostic and predictive biomarker in CRC. Indeed, antiangiogenic therapies (mainly based on the use of monoclonal antibodies against VEGF-A) [35] are administered, independently from evaluation of VEGF-A levels, to promote vessel normalization, a process that – restoring proper tumor perfusion and oxygenation – limits tumor cell invasiveness and improve the effectiveness of anticancer treatments [36–38].

Here we found that in CRC patients there is a positive correlation between TRF2 and VEGF-A and high levels of TRF2 confer prognostic value to VEGF-A, identifying a subclass of patients with higher risk of disease relapse/ progression.

## Methods

### Case selection

The study group comprised a retrospective series of 185 unselected patients surgically treated for colorectal adenocarcinoma at the Regina Elena National Cancer Institute, Rome, Italy, between January 2000 and December 2013. Clinical data were obtained from hospital medical records and included details pertaining to patient gender and age, tumor differentiation, location, size, TNM stage, lymph node (LN) metastasis, histopathological grade, and treatments. Tumors were staged according to Singh C. Staging of colonic carcinoma (AJCC 7th Edition) PathologyOutlines.com website (http://www.pathologyoutlines.com/topic/colontumorstaging.html - Accessed May 14th, 2020).

### Tissue microarray construction

For the purposes of this retrospective cross-sectional study, all colorectal cancers included in the study were histopathologically re-evaluated on haematoxylin and eosin stained slides and representative areas were marked prior to tissue microarray (TMA) construction.

Two core cylinders (1 mm diameter) were taken from the CRC samples and deposited into two separate recipient paraffin blocks using a specific arraying device (Alphelys, Euroclone, Milan, Italy).

In cases where informative results on TMA were absent due to missing tissue, no tumor tissue, or unsuccessful staining, we re-analyzed the correspondent routine tissue section. In addition to tumor tissues, the recipient block also received normal colon tissue as negative controls.

Two-μm sections of the resulting microarray block were made and used for immunohistochemical (IHC) analysis after transferring them to SuperFrost Plus slides (Menzel-Gläser, Braunschweig, Germany).

### Immunohistochemistry

Immunohistochemical (IHC) staining on TMA was performed using anti-TRF2 mouse monoclonal antibody (clone 4A794; Upstate, Sial, Rome, Italy) and anti-VEGF-A rabbit polyclonal antibody (Abcam Ltd., Cam- bridge, UK) in an automated immunostainer (Bond-III, Leica Biosystem, Milan, Italy). A pH 6 buffer was used as antigen retrieval for the two antibodies according to the manufacturer’s protocol. 3,3′-diaminobenizidine tetrahydrochloride (DAB) visualized TRF2 mouse antibody via a brown precipitate and Fast Red detected VEGF-A rabbit antibody via a red precipitate. Images were obtained at 20x magnification by using a light microscope equipped with a software able to capture images (DM2000 LED, Leica). The levels of TRF2, a telomere subunit localized in the nucleus, and VEGF-A, a cytoplasmic factor playing a key role in promoting angiogenesis, were evaluated in terms of intensity of nuclear (TRF2) and cytoplasmic (VEGF-A) staining, respectively (0 = negative, 1+ = weak, 2+ = moderate, 3+ = strong). Evaluation of the IHC results was performed independently and in blinded manner by two investigators. TRF2 and VEGF-A expression were scored semiquantitatively based on IHC staining intensity: low intensity cases displayed a 0/1+ IHC score and high intensity cases presented a 2+/3+ IHC score.

### Targeted DNA NGS

Genomic DNA was extracted on the QIAcube® platform using the QIAamp DNA FFPE tissue kit (Qiagen) according to the manufacturer’s instructions.

All DNA samples were then quantified by a Qubit Fluorometer (Termofisher Scientific, Waltham, Massachusetts, USA) using a Qubit® dsDNA HS Assay Kit. Library preparation was performed on 10 ng DNA (range from 1 to 20 ng) by the Ion AmpliSeq Library Kit 2.0 (Termofisher Scientific) and The Ion AmpliSeq™Cancer Hotspot Panel v2 (Termofisher Scientific) which generates 207 amplicons covering approximately 2800 COSMIC mutations in 50 different oncogenes and tumor suppressor genes: *ABL1, AKT1, ALK, APC, ATM, BRAF, CDH1, CDKN2A, CSF1R, CTNNB1, EGFR, ERBB2, ERBB4, EZH2, FBXW7, FGFR1, FGFR2, FGFR3, FLT3, GNA11, GNAS, GNAQ, HNF1A, HRAS, IDH1, JAK2, JAK3, IDH2, KDR, KIT, KRAS, MET, MLH1, MPL, NOTCH1, NMP1, NRAS, PDGFRA, PIK3CA, PTEN, PTPN11, RB1, RET, SMAD4, SMARCB1, SMO, SRC, STK11, TP53*, and *VHL*.

Each library was barcoded with the Ion Xpress Barcode Adapters 1–16 Kit (Termofisher Scientific) and diluted to a final concentration of 100 pM; barcoded libraries were pooled in equimolar amount and diluted to 35 pM for downstream template preparation. Template preparation was performed by the Ion Chef system (Termofisher Scientific), which integrates library amplification, Ion Sphere particles (ISP) recovery-enrichment and Chip loading. Sequencing was performed on Ion S5 system (Termofisher Scientific), with the Ion 530 chips. Raw data were analyzed using the Torrent Suite Software v.5.4. (Termofisher Scientific). The coverage analysis was performed using the coverage analysis plug-in v.5.4. Quality criteria used as end points were a detection threshold of 5% and a minimum coverage depth of 200x. Polymorphic variants were filtered out exploiting the Ion Reporter Suite (Termofisher Scientific). Only single nucleotide variants (SNVs) resulting in a nonsynonymous amino acid change, or a premature stop codon, and all short indels resulting in either a frameshift or insertion/deletion of amino acids were selected. All variants were manually reviewed with Integrative Genomics Viewer (IGV v.2.8.0, Broad Institute, Cambridge, Massachusetts, USA) and with the support of publically available datasets reporting on their established or predicted oncogenicity (i.e. COSMIC, cBioPortal, Clinical Trials, ClinVar). All molecular analyses were carried out in tissue samples collected before the administration of first-line chemotherapy for advanced disease.

### TCGA data analysis

Genomic/transriptomic data regarding patients in the TCGA cohort were extracted from cBioPortal (Colorectal Adenocarcinoma, TCGA - PanCancer Atlas; last accessed on 11th Feb 2020). Patients with missing data regarding disease stage, VEGF-A/TRF2 mRNA expression and time-to-event endpoints were excluded from the analysis. Samples with VEGF-A and TRF2 mRNA values greater than the median were classified either as VEGF-A high (VEGF-A^H^), TRF2 high (TRF2^H^). On the contrary, mRNA values lower than the median were classified as VEGF-A low (VEGF-A^L^) or TRF2 low (TRF2^L^). All combinations of TRF2/VEGF-A levels were evaluated. Survival analysis were performed with the Kaplan-Meier product-limit method from the date of surgery until the time of death for any cause (Disease specific survival – DSS, Disease-free Survival – DFS), The log-rank test was used to assess differences between subgroups. Significance was defined at the *p* ≤ 0.05 level.

### Statistical analysis

The associations between variables were tested by Pearson Chi Square test or Fisher Exact test, when appropriate. The Hazard Ratio and confidence limits (CI) were estimated for each variable using the Cox univariate model. Significance was defined at the *p* ≤ 0.05 level. A multivariate Cox hazard model was developed using stepwise regression (forward selection) by selecting significant variables upon univariate analysis. Enter limit and remove limit were *p* = 0.10 and *p* = 0.15, respectively. Potential markers of prognostic significance included: sex, age, stage, site, grading, tumor size, lymph-node, metastasis, VEGF-A, TRF2. Survival curves were calculated by the Kaplan–Meier method from the date of surgery until relapse or death for any cause (Disease Free Survival – DFS) or from the date of the surgery until progression or death for any cause (Progression Free Survival – PFS). Since our cohort included both stage I-III patients and metastatic (stage IV) patients, the outcome Progression/Disease Free Survival (P/DFS) was used. The log-rank test was used to assess differences between subgroups. Significance was defined at the *p* ≤ 0.05 level. SPSS software (SPSS version 21.0, S PSS Inc., Chicago, Illinois, USA) was used for statistical evaluations.

## Results

### Patient sample classification

In this study, we analysed paraffin-embedded tumor samples of 185 CRC patients treated at the Regina Elena National Cancer Institute of Rome. The study was reviewed and approved by the ethics committee of the Institute. Detailed clinical and pathological features of all cases are displayed in Table 1.

**Table 1:** Clinicopathological characteristics of evaluated CRC patients

Number of patients	185
**Tumor size**^**a**^	
1	2 (1.1%)
2	20 (10.8%)
3	119 (64.3%)
4	44 (23.8%)
**Lymph-node**	
Negative (N-)	83 (44.9%)
Positive (N+)	102 (55.1%)
**Distant metastasis**	
Negative (M0)	145 (78.4%)
Positive (M+)	40 (21.6%)
**Grading**	
1	2 (1.1%)
2	148 (80.0%)
3	35 (18.9%)
**Stage**	
I-II	74 (40%)
III	68 (36.8%)
IV	43 (23.2%)
**Age**	
Average	65 yrs
Minimum	35 yrs
Maximum	90 yrs
**Sex**	
Male	117 (63.2%)
Female	68 (36.8%)
**Site**	
Rectum	49 (26.5%)
Right colon	59 (31.9%)
Left colon	77 (34.0%)

^*a*^*Tumor size: T1 – tumor invades submucosa; T2 – tumor invades muscularis propria; T3 – tumor invades through the muscularis propria into the pericolorectal tissues; T4 – T4a: tumor penetrates to the surface of the visceral peritoneum, T4b: tumor directly invades or is adherent to other organs or structures*

Of note, data relative to treatments were available for 145 out 185 patients in our dataset. Of these, 82 received adjuvant therapy (Supplementary Table S1) whilst the remaining 63 did not receive treatment (mainly stage I and low risk stage II patients but also certain patients that for undefined reasons were not treated).

**Fig. 1:**
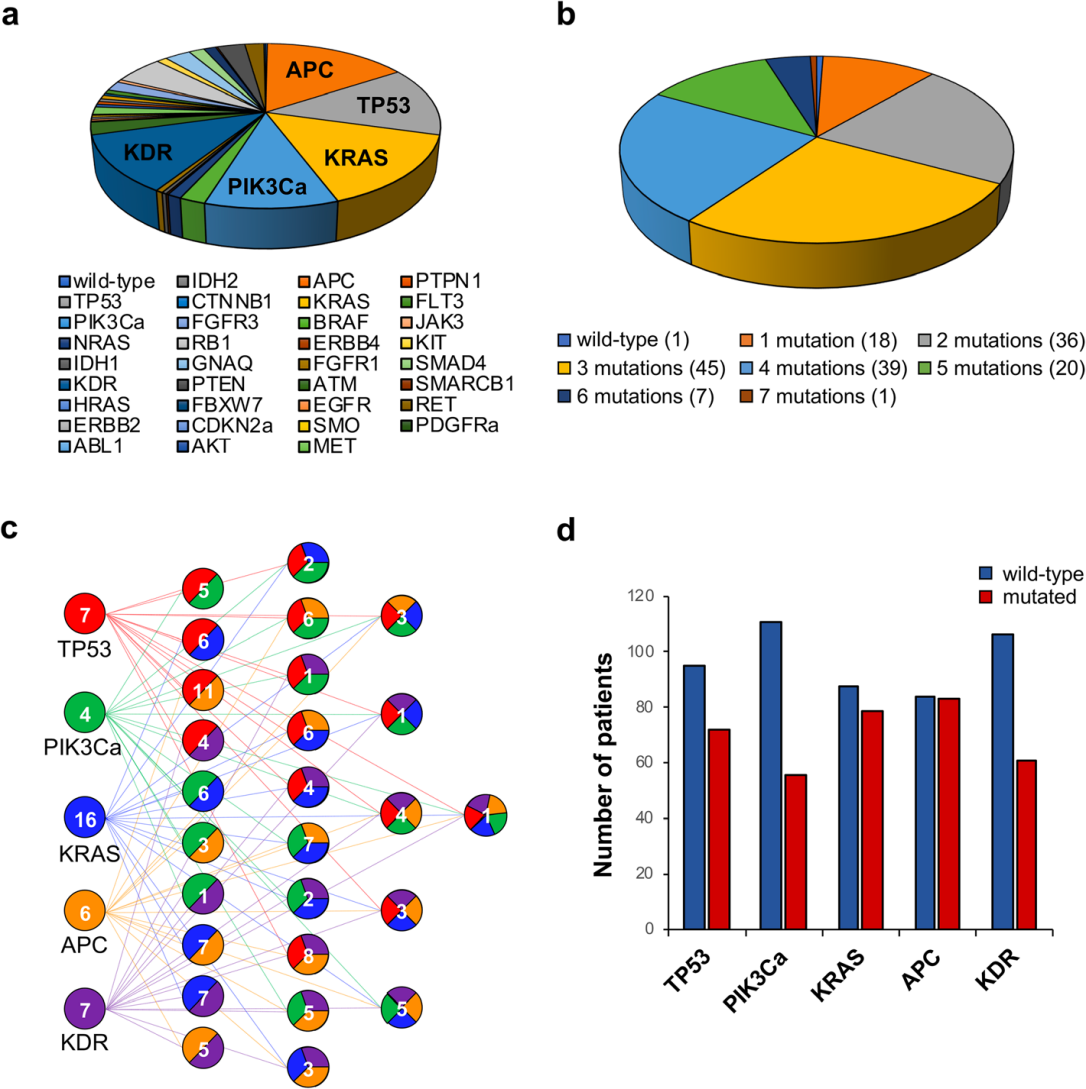
Mutational profile of CRC patients evaluated by NGS analysis. **a** Pie chart showing frequency of the mutations detected in tumor samples from the CRC patients evaluated in the study. **b** Pie chart showing distribution of patients according to the number of mutations (from 0 to 7) simultaneously detected for each sample. For each condition, the number of patients is reported in the brackets. **c** Scheme showing the distribution of patients carrying mutated forms of TP53 (red), PIK3Ca (green), KRAS (blue), APC (orange) or KDR (purple), singularly or in combination (from 2 to 5 simultaneous mutations). Number of patients is reported for each mutational profile. **d** Histograms showing the number of patients mutated (red bars) or wild-type (blue bars) for each evaluated gene (TP53, PIK3Ca, KRAS, APC or KDR)

Additionally, in order to establish the presence of genetic alterations, samples were characterized by Next Generation Sequencing (NGS) using a commercial targeted NGS panel of 50 genes known or highly suspected to promote various tumor types, included CRC (Fig. 1a and Supplementary Table S2). Of the 185 analysed cases, 18 were not evaluable for technical reasons, 1 was wild-type for all the investigated genes, 18 were mutated in a single gene, and the remaining 148 cases carried multiple gene mutations (from 2 to 7) (Fig. 1b), indicating that different mutated genes can coexist in a single sample. Moreover, a detailed data analysis evidenced that the most common mutations concerned APC, TP53, KRAS, PI3KCa and KDR (Fig. 1a). Among these 5 genes, APC, TP53, KRAS and PI3KCa are relevant in the tumorigenesis of CRC, whilst KDR encodes for VEGFR2, the main receptor VEGF-A, and its mutational state could be relevant in defining the prognostic role of VEGF-A in CRC patients. Notably, 156 out of 166 patients were mutated in the evaluated genes and, more precisely, 40 presented a single mutated gene while 116 showed multiple alterations (55 double, 44 triple, 16 quadruple and 1 quintuple mutations), as detailed in the (Fig. 1c) Finally, for each of the selected oncogenes, the patients were categorized, depending on the presence or absence of that specific mutation, into wild-type or mutated (Fig. 1d), evidencing that 49.7% were mutated for APC, 43.1% for TP53, 47.3% for KRAS, 33.5% for PI3KCa and 36.5% for KDR.

### Immunohistochemical evaluation of TRF2 and VEGF-A expression levels

**Fig. 2:**
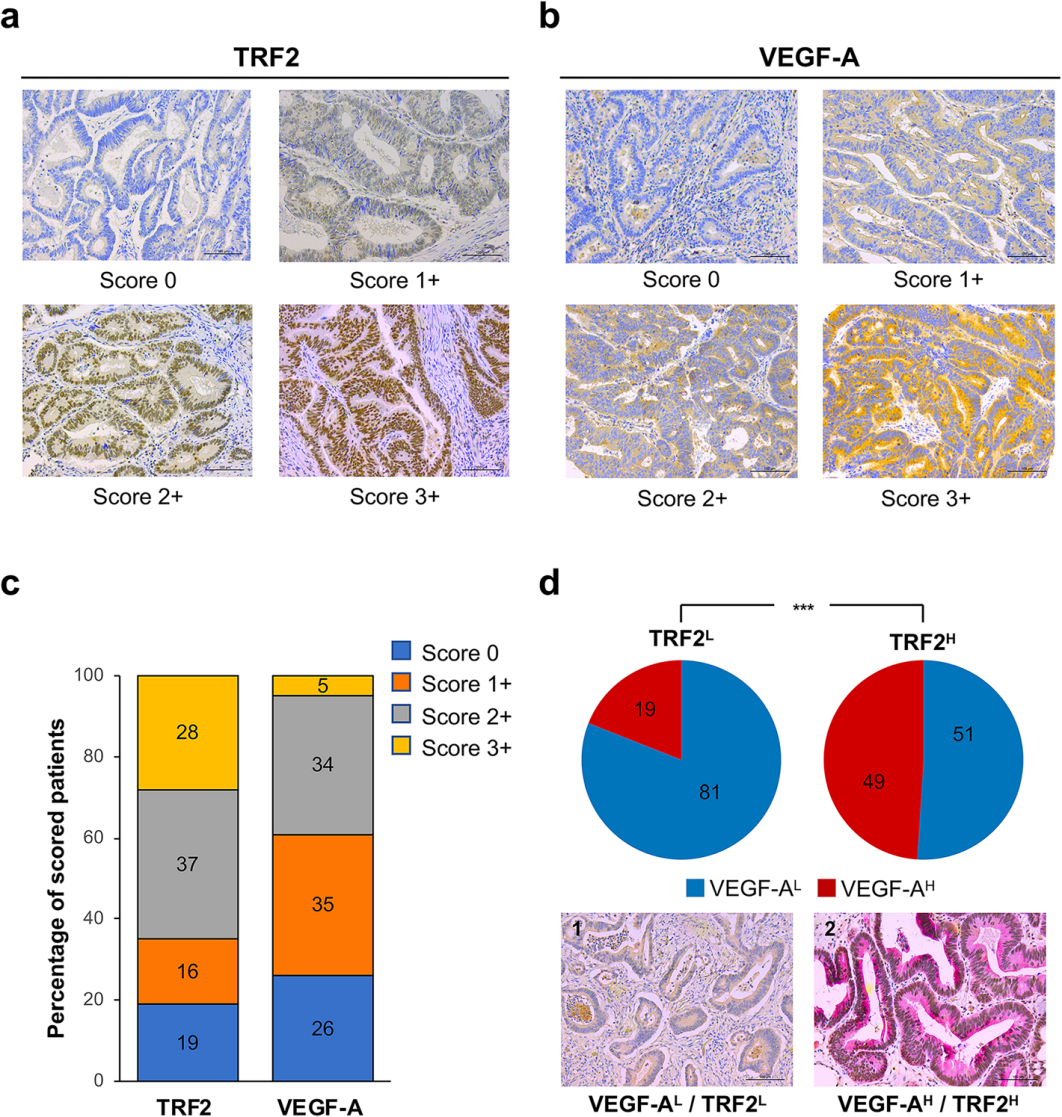
Association between TRF2 and VEGF-A expression in CRC patients. **a** and **b** Immunohistochemical (IHC) score of CRC samples labelled with the indicated antibodies. **a** Representative TMA sections stained for TRF2. Depending on the intensity of nuclear immunoreactivity, tumor samples were classified as negative (score 0), low (score 1+), medium (score 2+), or high (score 3+). **b** Representative images of CRC samples stained for VEGF-A. Samples’ classification was based on the intensity of the cytoplasmic immunostaining: negative (score 0), faint (score 1+), moderate (score 2+) or intense (score 3+). **c** Histogram showing the percentage of patients divided on the basis TRF2 and VEGF-A immuhistochemical scores, respectively. Score 0 (blue), 1+ (orange), 2+ (grey) and 3+ (yellow). **d ***Upper panel* - pie charts showing the distribution of VEGF-A low (VEGF-A^L^, score 0/1+) and VEGF-A high, (VEGF-A^H^, score 2+/3+) in the sub-populations of TRF2 low (TRF2^L^, score 0/1+) and TRF2 high (TRF2^H^, score 2+/3+) patients (***P < 0.001; χ^2^ test). *Lower panel* – IHC evaluation of TRF2 and VEGF-A expression in two representative CRC samples showing (1) low levels of VEGF-A and TRF2 (VEGF-A^L^/TRF2^L^) and (2) high levels of VEGF-A and TRF2 (VEGF-A^H^/TRF2^H^). Scale bar: 100 μm

TMA sections of 185 CRC samples were labelled with antibodies against TRF2 and VEGF-A and scored, depending on the intensity of the staining (Fig. 2a, b). Colorectal adenocarcinomas were considered positive for TRF2 when neoplastic cells showed nuclear immunoreactivity. IHC staining was classified as negative, score 0; low, score 1+; medium, score 2+; and high, score 3+. Concerning VEGF-A, tumors exhibiting a detectable, but faint cytoplasmic immunostaining were scored as 1+, tumors displaying a complete cytoplasmic immunostaining with a moderate intensity were scored as 2+, whereas colorectal cancers showing a distinct and intense cytoplasmic immunostaining were scored as strongly positive 3+. For all the subsequent analyses, score 0/1+ was defined as low intensity expression (L) and score 2+/3+ as high intensity expression (H). In cases where informative IHC results on TMA were absent due to missing tissue or no tumor tissue, correspondent routine tissue sections were re-analyzed.

Notably, of the entire series of 185 samples, 121 (65%) CRC demonstrated a high TRF2 expression (TRF2^H^, score 2+/3+), while high expression of VEGF-A (VEGF-A^H^, score 2+/3+) was observed in 71 (39%) patients (Fig. 2c and Supplementary Table S3). Moreover, as reported in the (Fig. 2d), among the 64 tumors with low TRF2 levels (TRF2^L^, score 0/1+), 52 (81%) were VEGF-A^L^ (score 0/1+) while the remaining 12 cases (19%) were VEGF-A^H^. Conversely, of 121 TRF2^H^ CRCs, 62 (51%) were VEGF-A^L^ and 59 (49%) VEGF-A^H^. χ^2^ test evidenced a significant association between the two parameters (*p* < 0.0001; Fig. 2d).

### Clinical relevance of TRF2 and VEGF-A association

**Fig. 3:**
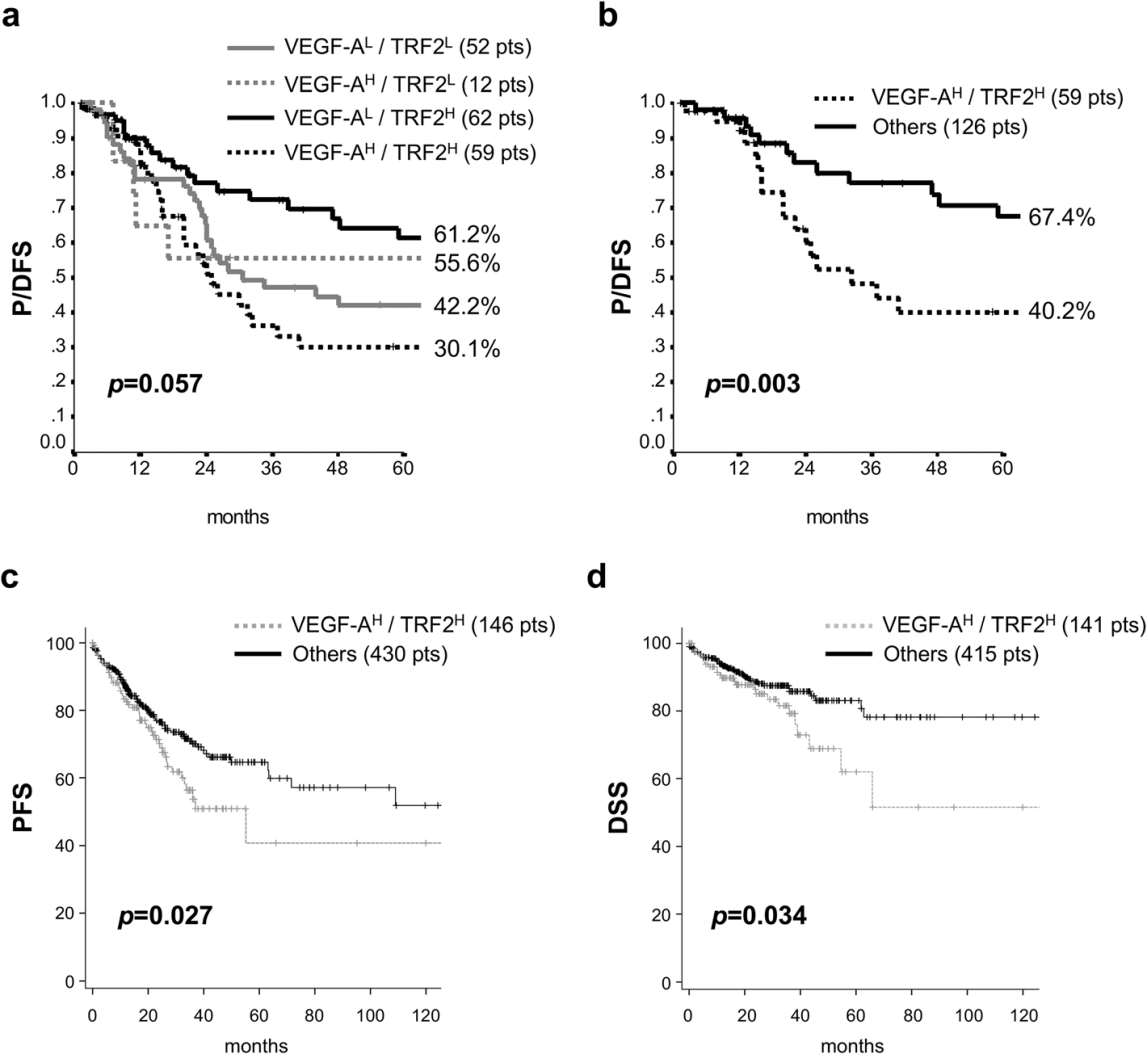
Clinical outcome of CRC patients stratified on the basis of VEGF-A and TRF2 levels. **a** and **b** Progression/ Disease Free Survival evaluated by Kaplan–Meier curves on the panel of 185 CRC patients retrospectively evaluated in our institute. **a** Patients were stratified on the basis of VEGF-A and TRF2 expression and survival was evaluated in patients’ subgroups with all the possible combinations of VEGF-A/TRF2 levels: VEGF-A high/ TRF2 high (VEGF-A^H^/TRF2^H^), VEGF-A high/ TRF2 low (VEGF-A^H^/TRF2^L^), VEGF-A low/ TRF2 high (VEGF-A^L^/TRF2^H^), VEGF-A low/ TRF2 low (VEGF-A^L^/TRF2^L^). **b** Patients were stratified as in (**a**) and survival was evaluated by comparing patients expressing high levels of VEGF-A and TRF2 (VEGF-A^H^/TRF2^H^) with all the others patients’ subgroups (others). For each sub-population, the number of patients is reported in the brackets. Percentages of surviving patients are reported close to the respective curves. **c** Progression-free survival (PFS) and **d** Disease Specific Survival evaluated by Kaplan–Meier curves on CRC patients from the TCGA dataset. Survival of patients, stratified on the basis of TRF2 and VEGF-A mRNA expression, was evaluated as in (**b**)

**Fig. 4:**
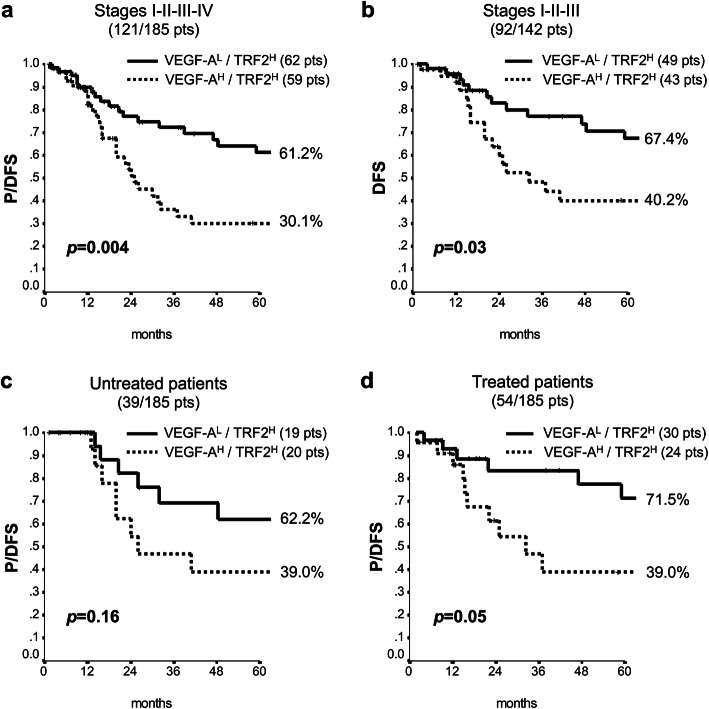
Impact of the direct correlation between TRF2 and VEGF-A on patients’ survival. Survival analysis of CRC patients stratified on the basis of the levels of VEGF-A and TRF2. **a** Progression/ Disease Free Survival of patients expressing high levels of VEGF-A and TRF2 (VEGF-A^H^/TRF2^H^) was compared with that of patients in which high levels of TRF2 correlate with low VEGF-A expression (VEGF-A^L^/TRF2^H^). **b** Disease Free Survival of stage I-III CRC patients. **c** and **d** Progression/ Disease Free Survival of the VEGF-A^H^/TRF2^H^ and VEGF-A^L^/TRF2^H^ patients who (**c**) did not receive (Untreated patients) or (**d**) did receive (Treated patients) adjuvant therapy. For each sub-population the number of patients is reported in the brackets. Percentages of surviving patients are reported close to the respective curves

In order to establish the prognostic value of TRF2 and VEGF-A, the 185 patients, with a median follow-up of 66 months (95% CI 61.8–71.5), were retrospectively evaluated for progression/disease-free survival (P/DFS). Interestingly, when the patients were analyzed by Kaplan-Meier curves, we observed that, whilst differences in TRF2 levels did not affect the outcome of interest (*p* = 0.60), high expression levels of VEGF-A (VEGF-A^H^) identified a subgroup of patients at higher risk of relapse/progression (*p* = 0.04) (Supplementary Fig. S1a, b). Concerning the effects of VEGF-A/TRF2, we noticed that patients expressing high levels of both VEGF-A and TRF2 (VEGF-A^H^/TRF2^H^) were characterised by shorter P/DFS (Fig. 3a). Of note, four-arms analysis revealed a borderline significant association (*p* = 0.057), which became fully significant when VEGF-A^H^/TRF2^H^ patients were compared with all the other biomarker combinations (Fig. 3b; *p* = 0.003). Interestingly these data were confirmed also in a larger cohort of 621 CRC patients from the The Cancer Genome Atlas dataset (TCGA; 10.7908/C11G0KM9), whose clinical outcome was evaluated in terms of both Progression Free Survival (PFS) and Disease Specific Survival (DSS) (Fig. 3c, d). Moreover, clinical relevance of VEGF-A (Supplementary Fig. S1b) is exacerbated by high levels of TRF2 expression, as demonstrated by the reduced probability of survival of the VEGF-A^H^/TRF2^H^ patients compared with the VEGF-A^L^/TRF2^H^ ones (Figs. 3a and 4a). Notably, these results assumed still more interest when stage IV were excluded from the analysis (Supplementary Table S4, S5, S6). Indeed, while TRF2 and VEGF-A alone had no effect on DFS (Supplementary Fig. S1c, d), their combination maintained prognostic significance (*p* = 0.03) in tumors in which high VEGF-A was associated with high TRF2 expression levels (Fig. 4b). Next, we estimated P/DFS in the subset of patients (*N* = 145) for whom data related to administered treatments and outcomes were available. Interestingly, Kaplan-Meier curves (Fig. 4c, d and Supplementary Fig. S2) showed that administration of adjuvant therapy – mainly FOLFOX and 5-FU (Supplementary Table S1), two treatments that are not reported to affect telomere biology or angiogenic response – produced a beneficial effect on VEGF-A^L^/TRF2^H^ patients but not on the VEGF-A^H^/TRF2^H^ ones, suggesting that this latter group of patients might benefit of combinatorial treatment with common adjuvant therapies and anti-VEGF-A drugs.

Finally, in the univariate analysis (Cox model), tumor site (HR 1.86, CI 1.18–2.95, *p* = 0.08), pN (HR 1.63, CI 1.04–2.57, *p* = 0.03), pM (HR 2.89, CI 1.79–4.68, *p* < 0.0001), VEGF-A (HR 1.58, CI 1.01–2.48, *p* = 0.046), and the combination VEGF-A^H^/TRF2^H^ (HR 1.66, CI 1.04–2.64, *p* = 0.032) were associated with an increased risk of disease recurrence (Table 2).

**Table 2:** Univariate analysis

Number of patients	185
Variables	HR (95% CI; *p* value)
**Age***(> 67* vs *≤ 67)*	1.01 (0.65–1.57; *p* = 0.97)
**Sex***(male* vs *female)*	1.35 (0.84–2.17; *p* = 0.22)
**Site***(rectum* vs *colon)*	1.86 (1.18–2.95; *p* = 0.08)
**Size***(3/4* vs *1/2)*	1.50 (0.72–3.12; *p* = 0.28)
**Node***(N+* vs *N-)*	1.63 (1.04–2.57 *p* = 0.03)
**Metastasis***(M+* vs *M-)*	2.89 (1.79–4.68; *p <* 0.0001)
**Grading***(3* vs *1/2)*	1.25 (0.72–2.17; *p* = 0.42)
**VEGF-A***(L* vs *H)*	1.58 (1.01–2.48; *p* = 0.046)
**TRF2***(L* vs *H)*	1.13 (0.72–1.77; *p* = 0.60)
**VEGF-A/TRF2***(H/H* vs *others)*	1.66 (1.04–2.64; *p* = 0.032)

Moreover, tumor site, metastatic disease and VEGF-A were confirmed as prognostic factors in multivariate analysis (Cox model) (Table 3). In particular, VEGF-A was found to be an independent predictor of adverse outcomes also in that subclass of patients expressing high levels of TRF2 expression (HR 1.93, CI 1.05–3.55, *p* = 0.03) (Table 3).

**Table 3:** Multivariate analysis

Variables	TOTAL SAMPLES (185 patients)	TRF2^H^ (122 patients)	TRF2^L^ (63 patients)
	HR (95% CI; *p* value)		
**Site***(rectum* vs *colon)*	1.90 (1.19–3.03; *p* = 0.007)	2.15 (1.17–3.93; *p* = 0.013)	–
**Metastasis***(M+* vs *M-)*	3.05 (1.88–4.96; *p* = 0.0001)	3.70 (1.96–6.98; *p* < 0.0001)	2.25 (1.04–4.88; *p =* 0.04)
**VEGF-A***(H* vs *L)*	1.49 (0.94–2.36; *p* = 0.087)	1.93 (1.05–3.55; *p* = 0.03)	–

Altogether, these data support the idea that even if TRF2 has not prognostic relevance per se, an increase of its levels exacerbate the already negative clinical outcome associated with high levels of VEGF-A expression.

Since CRC pathogenesis, drug responsiveness and clinical outcome have been reported to also depend on the anatomical location of the tumor and can differ between right side and left side of the colon [39], we evaluated the prognostic relevance of TRF2 and VEGF-A by distinguishing colon cancer between right- and left-sided. Probably due to the limited number of available patients (Table 1), we were unable to observe any significant difference in the survival rate between patients with right-sided and left-sided colon cancer (HR 1.28; 0.73–2.25; *p* = 0.39). Moreover, we did not observe any significant interaction (*p* = 0.91) between the tumor site and the expression levels of TRF2 and/or VEGF-A.

Next, on the basis of the NGS analysis (Fig. 1), it was also evaluated whether survival associated with the levels of VEGF-A and TRF2 was or not dependent on the mutational state of APC, TP53, KRAS, PI3KCa and KDR. Interestingly, by stratifying the patients on the basis of TRF2 (TRF2^L^ vs TRF2^H^) or VEGF-A (VEGF-A^L^ vs VEGF-A^H^), we did not find statistically significant correlations between the levels of these two variables and the mutations of TP53, PI3KCa, KRAS, APC and KDR (Supplementary Fig. S3). Despite these results and the very limited sample sizes available, the analyses revealed that the mutational state of the evaluated genes did not affect the prognostic value of TRF2 and VEGF-A, evaluated both singularly (supplementary Fig. S4, S5, S6, S7, S8) or in combination (Supplementary Fig. S9-S10). In particular, high levels of VEGF-A and TRF2 expression (VEGF-A^H^/TRF2^H^) always correlates with a poor prognosis (Supplementary Fig. S9–10), confirming and reinforcing the predictive relevance of combinatorial analyses based on the evaluation of VEGF-A and TRF2 expression levels in CRC patients, independently from the mutational state of the evaluated driver oncogenes and/or the VEGF-A receptor. The results obtained in our patients were then confirmed also in the patients from the TCGA dataset (Supplementary Fig. S11–12). For completeness, the analyses were also extended to BRAF. Indeed, even if the mutational rate of this gene is quite low in CRC patients (about 7–10%), BRAF plays a critical role in the prognosis and for this reason its role would be relevant in our study [40, 41]. Due to the limited number of patients carrying the mutated form of BRAF in our dataset (only 11/185 patients, as reported, see Supplementary Table S2), the analyses were directly performed in patients from the TCGA. Notably, the results demonstrated that the prognostic value of TRF2/VEGF-A association is not affected by the mutational state of BRAF (Supplementary Fig. S13), definitively confirming the role of TRF2/VEGF-A as independent prognostic factors in CRC patients.

## Discussion

CRC is one of the leading causes of mortality and morbidity in developed countries. To date, prediction of clinical outcome of CRC patients is only based on the evaluation of tumor stage, lymph-node positivity and presence of distant metastases. However, some patients, classified as low-risk subjects, develop local recurrence or metastasis years after receiving surgical treatment [42], evidencing the urgent needed of identifying novel and more effective prognostic markers. In the last few times there has been a rapid growth in the number of clinical studies aimed at identifying biomarkers able to discriminate the patients that might take a real advantage from administration of therapies beyond the surgical treatment. In this scenario, telomeres were evaluated as a putative prognostic factor in CRC, even if lack of solid evidences and a limited amount of available data raise many doubts about their effective clinical relevance [15, 16, 21]. Conversely, the telomeric proteins have been poorly investigated in clinical studies and very little is still known about their role as tumor biomarkers [43, 44].

Here, based on previous studies showing that TRF2 is over-expressed in CRC [29, 32, 33] and it is a marker of poor prognosis in several tumor histotypes [45, 46], we assayed the predictive role of TRF2 on the outcome of CRC patients. Notably, Kaplan-Meier and Cox regression analyses, performed on a cohort of 185 CRC patients from our institute, evidenced that TRF2 is not an independent predictor of recurrence and prognosis for patients affected by this tumor. Despite the knowledges regarding TRF2 have been long limited to the sphere of telomere biology, it is now universally accepted that TRF2 also exerts telomere-unrelated functions also correlated with tumor formation and progression [24, 45, 47, 48]. In particular, data from our and other laboratories evidenced a role of TRF2 in controlling tumor angiogenesis [33, 34, 49]. Since its discovery, angiogenesis – the process of vessel formation from stromal cells – has been identified as a critical event for promoting tumor growth and metastasis [50–52]. Among the several factors participating in angiogenesis, VEGF-A has been extensively explored as prognostic marker, but its relevance in predicting the outcome of CRC patients is quite controversial. Indeed, while some studies show an association between over-expression of VEGF-A and poor CRC outcomes [53–55], others demonstrate that either VEGF-A has no significant prognostic value in CRC patients [42, 56–58] or it assumes prognostic relevance only in association with other factors [59, 60]. In accordance with these data, our results demonstrated that even if VEGF-A is a poor prognostic factor in CRC patients, its clinical relevance is lost when stage IV patients, a subclass of patients with a poor clinical outcome, are excluded from the survival analyses. Starting from these data, we evaluated the existence of a clinically-relevant association between TRF2 and VEGF-A. Interestingly, immunohistochemical evaluation of tumor samples evidenced the existence of a positive correlation between TRF2 and VEGF-A. Moreover, analysis of patients’ survival demonstrated that high levels of TRF2 and VEGF-A expression identify the stage I-III patients with a higher risk of relapse/progression. These results, corroborated by additional studies performed on a larger panel of CRC patients from the TCGA dataset, suggest that high levels of TRF2 expression impacts on VEGF-A exacerbating its prognostic relevance. Moreover, uni- and multi-variate analyses demonstrated that the association between high levels of TRF2 and VEGF-A represents, together with the tumor site and metastasis, a statistically relevant prognostic parameter in the evaluation of CRC patients. Of note, patients’ samples available for this study were also subjected to molecular profiling by NGS. The analyses, performed on a commercial targeted NGS panel of 50 genes, evidenced that APC, TP53, KRAS, PI3KCa and KDR were most frequently mutated in our patients but, regardless the relevance of these alterations in CRC, the mutational state of these genes did not affect the prognostic value of TRF2/VEGF-A, strengthening their clinical relevance.

Finally, analysing the effects of adjuvant therapies on the clinical outcomes, we noticed that patients expressing high levels of TRF2, have a benefit of chemotherapy only in the presence of low levels of VEGF-A, a situation that could be pharmacologically recapitulated through the administration of VEGF-A inhibitors to the patients expressing high levels of both TRF2 and VEGF-A. These data, although requiring further investigations, suggest that TRF2 – besides improving the prognostic value of VEGF-A – might be used, together with the VEGF-A, to identify a sub-group of patients that, independently from the mutational state of KDR (the gene encoding for VEGFR2), could take advantage from anti-angiogenic target-therapy. In this regard, experimental data produced on xenograft mice evidenced that treatment with the VEGF-A inhibitor bevacizumab determines a reduction of about the 50% in the growth of TRF2 over-expressing tumors (data not shown). Our study was carried out on 185 patients available in our institute and confirmed in a larger cohort of CRC patients from TCGA dataset. However, enlargement of our study through the enrolment and the subsequent prospective evaluation of new patients would be desirable.

## Conclusions

In conclusion, the results of this study permitted to identify TRF2 and VEGF-A association as a novel biomarker with prognostic relevance in CRC. In particular, co-expression of TRF2 and VEGF-A correlates with a poor clinical outcome in CRC patients identifying a subset of patients (mainly stage II and III) at higher risk of disease relapse/progression that could take an effective advantage from specific therapeutic regimens, included pharmacological approaches based on administration of angiogenic inhibitors.

## Supplementary information

**Additional file 1:Supplementary Table S1.** Type of adjuvant therapy administered to patients

**Additional file 2:Supplementary Table S2.** List of mutations detected by NGS analysis in our patients’ dataset

**Additional file 3:Supplementary Table S3.** Levels of TRF2 and VEGF-A evaluated on a cohort of 185 CRC patients

**Additional file 4:Supplementary Table S4.** Levels of TRF2 evaluated on staged CRC patients

**Additional file 5:Supplementary Table S5.** Levels of VEGF-A evaluated on staged CRC patients

**Additional file 6:Supplementary Table S6.** Combinatorial levels of TRF2 and VEGF-A evaluated on staged CRC patients

**Additional file 7:** **Supplementary Fig. S1.** Impact of TRF2 and VEGF-A levels on the survival of CRC patients

**Additional file 8:** **Supplementary Fig. S2.** Impact of TRF2 and VEGF-A levels on the response of CRC patients to adjuvant therapy

**Additional file 9:** **Supplementary Fig. S3.** Correlation between the levels of TRF2 and VEGF-A and the mutational state of TP53, PI3KCa, KRAS and APC

**Additional file 10:Supplementary Fig. S4.** Impact of TRF2 and VEGF-A levels on the survival of CRC patients, depending on the mutational state of TP53

**Additional file 11:** **Supplementary Fig. S5.** Impact of TRF2 and VEGF-A levels on the survival of CRC patients, depending on the mutational state of PIK3Ca

**Additional file 12:** **Supplementary Fig. S6.** Impact of TRF2 and VEGF-A levels on the survival of CRC patients, depending on the mutational state of KRAS

**Additional file 13.** **Supplementary Fig. S7.** Impact of TRF2 and VEGF-A levels on the survival of CRC patients, depending on the mutational state of APC

**Additional file 14:Supplementary Fig. S8.** Impact of TRF2 and VEGF-A levels on the survival of CRC patients, depending on the mutational state of KDR

**Additional file 15:** **Supplementary Fig. S9.** Imapct of the association of TRF2 and VEGF-A on clinical outcome of CRC patients, depending on the mutational state of TP53, PI3KCa, KRAS, APC and KDR

**Additional file 16:** **Supplementary Fig. S10.** Impact of the association of TRF2 and VEGF-A on clinical outcome of stage I-III CRC patients, depending on the mutational state of TP53, PI3KCa, KRAS, APC and KDR

**Additional file 17:Supplementary Fig. S11.** Impact of the association of TRF2 and VEGF-A on clinical outcome of CRC patients, depending on the mutational state of TP53, PI3KCa, KRAS, APC and KDR

**Additional file 18:** **Supplementary Fig. S12. ** Impact of the association of TRF2 and VEGF-A on clinical outcome of CRC patients, depending on the mutational state of TP53, PI3KCa, KRAS, APC and KDR

**Additional file 19:** **Supplementary Fig. S13.** Impact of the association of TRF2 and VEGF-A on clinical outcome of CRC patients, depending on the mutational state of BRAF

## Data Availability

The datasets used and or analysed during the current study are available from the corresponding authors on reasonable request.

## Abbreviations

CRC: Colorectal cancer
5-FU: 5-fluorouracil
VEGF: Vascular endothelial growth factor
EGFR: Epidermal growth factor receptor
DDR: DNA damage response
TRF2: Telomere Repeat binding Factor 2
TMA: Tissue microarray
IHC: Immunohistochemistry
DAB: 3,3′-diaminobenizidine tetrahydrochloride
LN: Lymph node
ISP: Ion sphere particles
SNVs: Single nucleotide variants
NGS: Next generation sequencing
PFS: Progression-free survival
DSF: Disease-free survival
DSS: Disease specific survival
TCGA: The Cancer Genome Atlas
HR: Hazard ratio
CI: Confidence limits
