# Supplementary material for: TRF2 and VEGF-A: an unknown relationship with prognostic impact on survival of colorectal cancer patients
Source: J Exp Clin Cancer Res. 2020 Jun 15;39:111. doi: 10.1186/s13046-020-01612-z (PMC7294609; doi:10.1186/s13046-020-01612-z)
Supplement: Supplementary file 1 — Additional file 1:Supplementary Table S1. Type of adjuvant therapy administered to patients [file 13046_2020_1612_MOESM1_ESM.docx]

**Supplementary Table S1** – Type of adjuvant therapy administered to patients

| **Total number of treated patients** | | | | | **82** |
| --- | --- | --- | --- | --- | --- |
|  |  |  |  |  |  |
|  |  |  |  | |  |
| **Administered adjuvant therapy** | | | | **Number of treated patients** | |
| **FOLFOX**  (folic acid + 5FU + oxaliplatin) | | | | **39** | |
| **FOLFOX + radiotherapy** | | | | **1** | |
| **De Gramont**  (folic acid + 5FU) | | | | **18** | |
| **De Gramont / 5FU + anastrozole** | | | | **1** | |
| **radiotherapy + capecitabine** | | | | **2** | |
| **capecitabine** | | | | **5** | |
| **FOLFOX / De Gramont** | | | | **4** | |
| **radiotherapy + 5FU / De Gramont** | | | | **1** | |
| **irinotecan + 5FU + cetuximab** | | | | **1** | |
| **radiotherapy** | | | | **3** | |
| **radiotherapy + De Gramont** | | | | **2** | |
| **FOLFOX + panitumumab** | | | | **1** | |
| **bevacizumab + dexamethasone + ranitidine** | | | | **1** | |
| **panitumumab + FOLFOX + radiotherapy** | | | | **1** | |
| **Bicalutamide*** | | | | **1** | |
| **not specified**** | | | | **1** | |
|  | | | | | |

* patient treated for the concomitant presence of prostatic adenocarcinoma.

** details regarding the administered therapy were missing in the hospital record.
