# Supplementary material for: TRF2 and VEGF-A: an unknown relationship with prognostic impact on survival of colorectal cancer patients
Source: J Exp Clin Cancer Res. 2020 Jun 15;39:111. doi: 10.1186/s13046-020-01612-z (PMC7294609; doi:10.1186/s13046-020-01612-z)
Supplement: Supplementary file 3 — Additional file 3:Supplementary Table S3. Levels of TRF2 and VEGF-A evaluated on a cohort of 185 CRC patients [file 13046_2020_1612_MOESM3_ESM.docx]

**Supplementary Table S3 –** Levels of TRF2 and VEGF-A evaluated on a cohort of 185 CRC patients.

| **Number of patients** | | **185** | |
| --- | --- | --- | --- |
|  |  |  |  |
|  | | |  |
| **TRF2 expression** | | | |
| 0 | 35 (19%) | | |
| 1+ | 29 (16%) | | |
| 2+ | 68 (37%) | | |
| 3+ | 53 (28%) | | |
|  | | | |
| **VEGF-A expression** | | | |
| 0 | 49 (26%) | | |
| 1+ | 65 (35%) | | |
| 2+ | 62 (34%) | | |
| 3+ | 9 (5%) | | |
