# Supplementary material for: TRF2 and VEGF-A: an unknown relationship with prognostic impact on survival of colorectal cancer patients
Source: J Exp Clin Cancer Res. 2020 Jun 15;39:111. doi: 10.1186/s13046-020-01612-z (PMC7294609; doi:10.1186/s13046-020-01612-z)
Supplement: Supplementary file 4 — Additional file 4:Supplementary Table S4. Levels of TRF2 evaluated on staged CRC patients [file 13046_2020_1612_MOESM4_ESM.docx]

**Supplementary Table S4** – Levels of TRF2 evaluated on staged CRC patients

| **Stage** | **TRF2^L^** | **TRF2^H^** | **Total** |
| --- | --- | --- | --- |
| **I-II** | 29  (39.2%) | 45  (60.8%) | 74  (100%) |
| **III** | 21  (30.9%) | 47  (69.1%) | 68  (100%) |
| **IV** | 14  (32.6%) | 29  (67.4%) | 43  (100%) |
| **Total** | 64  (34.6%) | 121  (65.4%) | 185  (100%) |
