# Supplementary material for: TRF2 and VEGF-A: an unknown relationship with prognostic impact on survival of colorectal cancer patients
Source: J Exp Clin Cancer Res. 2020 Jun 15;39:111. doi: 10.1186/s13046-020-01612-z (PMC7294609; doi:10.1186/s13046-020-01612-z)
Supplement: Supplementary file 5 — Additional file 5:Supplementary Table S5. Levels of VEGF-A evaluated on staged CRC patients [file 13046_2020_1612_MOESM5_ESM.docx]

**Supplementary Table S5** – Levels of VEGF-A evaluated on staged CRC patients

| **Stage** | **VEGF-A^L^** | **VEGF-A^H^** | **Total** |
| --- | --- | --- | --- |
| **I-II** | 45  (60.8%) | 29  (39.2%) | 74  (100%) |
| **III** | 45  (66.2%) | 23  (33.8%) | 68  (100%) |
| **IV** | 24  (55.8%) | 19  (44.2%) | 43  (100%) |
| **Total** | 114  (61.6%) | 71  (38.4%) | 185  (100%) |
