# Supplementary material for: TRF2 and VEGF-A: an unknown relationship with prognostic impact on survival of colorectal cancer patients
Source: J Exp Clin Cancer Res. 2020 Jun 15;39:111. doi: 10.1186/s13046-020-01612-z (PMC7294609; doi:10.1186/s13046-020-01612-z)
Supplement: Supplementary file 6 — Additional file 6:Supplementary Table S6. Combinatorial levels of TRF2 and VEGF-A evaluated on staged CRC patients [file 13046_2020_1612_MOESM6_ESM.docx]

**Supplementary Table S6** –Combinatorial levels of TRF2 and VEGF-A evaluated on staged CRC patients.

| **Stage** | **VEGF-A^L^/TRF2^L^** | **VEGF-A^L^/TRF2^H^** | **VEGF-A^H^/TRF2^L^** | **VEGF-A^H^/TRF2^H^** | **Total** |
| --- | --- | --- | --- | --- | --- |
| **I-II** | 22  (29.7%) | 23  (31.1%) | 7  (9.5%) | 22  (29.7%) | 74 (100%) |
| **III** | 19  (27.9%) | 26  (38.2%) | 2  (2.9%) | 21  (30.9%) | 68  (100%) |
| **IV** | 11  (25.6%) | 13  (30.2%) | 3  (7%) | 16  (37.2%) | 43  (100%) |
| **Total** | 52  (28.1%) | 62  (33.5%) | 12  (6.5%) | 59  (31.9%) | 185  (100%) |
