# Supplementary figures and images for: TRF2 and VEGF-A: an unknown relationship with prognostic impact on survival of colorectal cancer patients
Source: J Exp Clin Cancer Res. 2020 Jun 15;39:111. doi: 10.1186/s13046-020-01612-z (PMC7294609; doi:10.1186/s13046-020-01612-z)

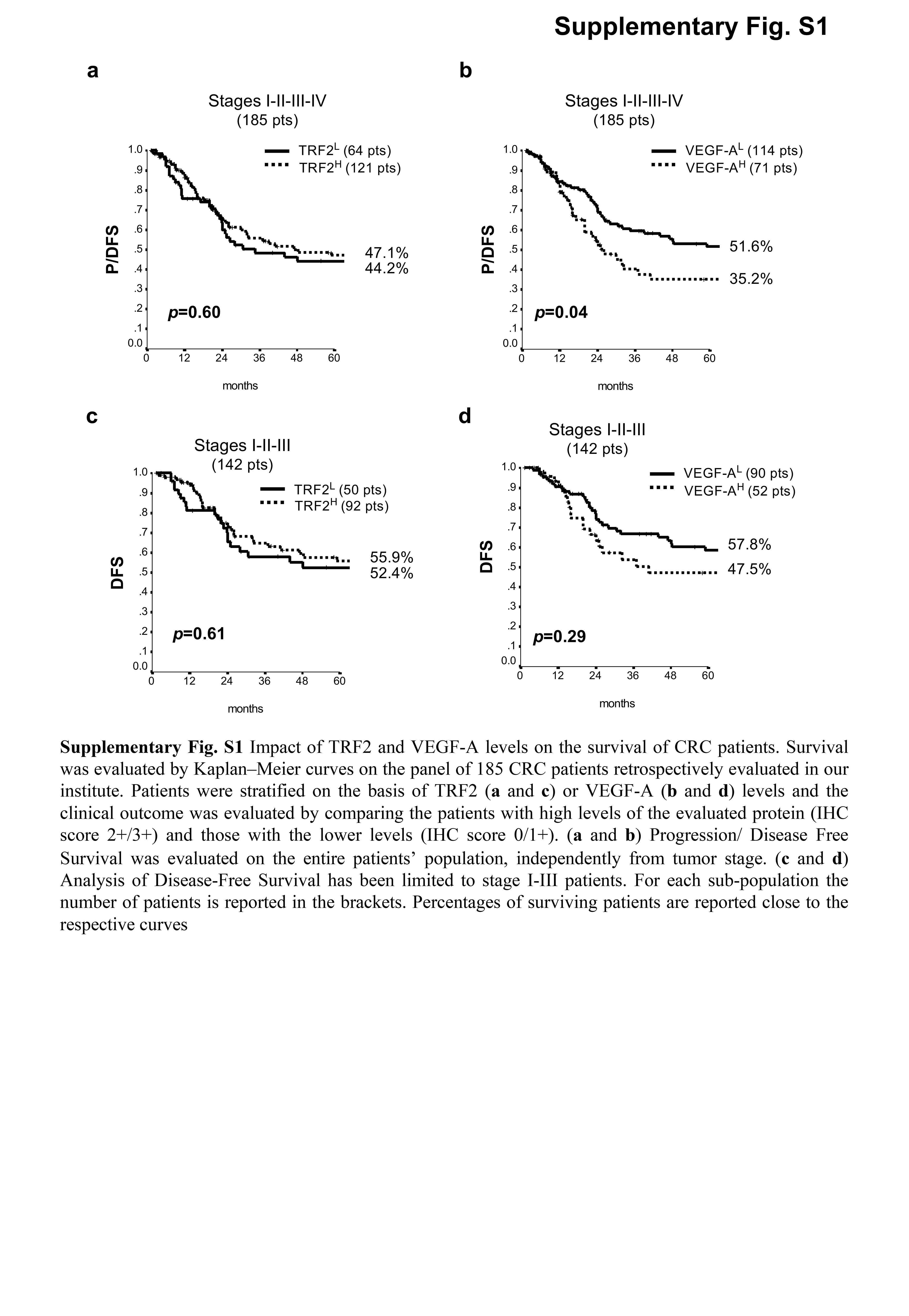

Supplement: Supplementary file 7 — Additional file 7: Supplementary Fig. S1. Impact of TRF2 and VEGF-A levels on the survival of CRC patients [file 13046_2020_1612_MOESM7_ESM.tif]

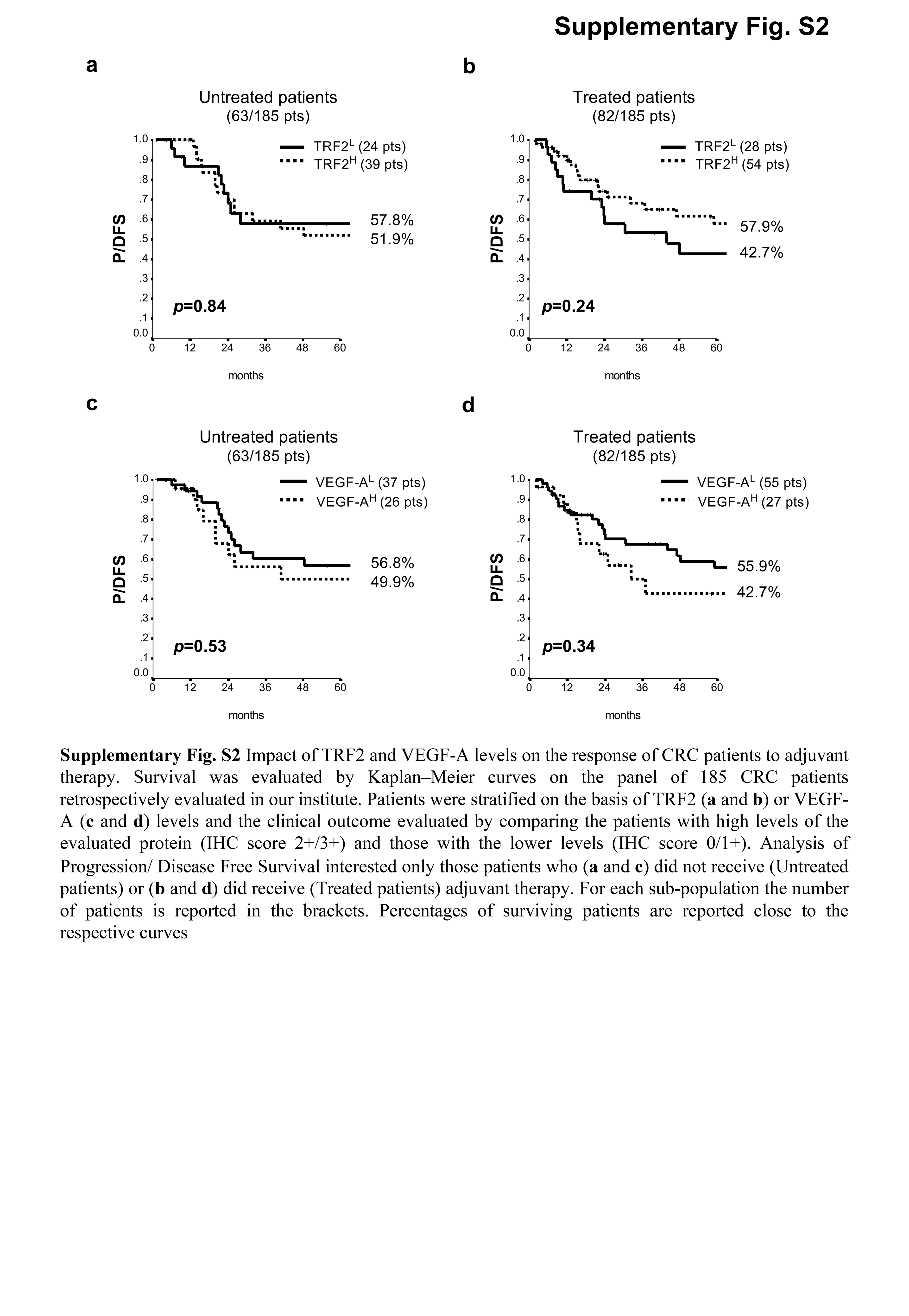

Supplement: Supplementary file 8 — Additional file 8: Supplementary Fig. S2. Impact of TRF2 and VEGF-A levels on the response of CRC patients to adjuvant therapy [file 13046_2020_1612_MOESM8_ESM.tif]

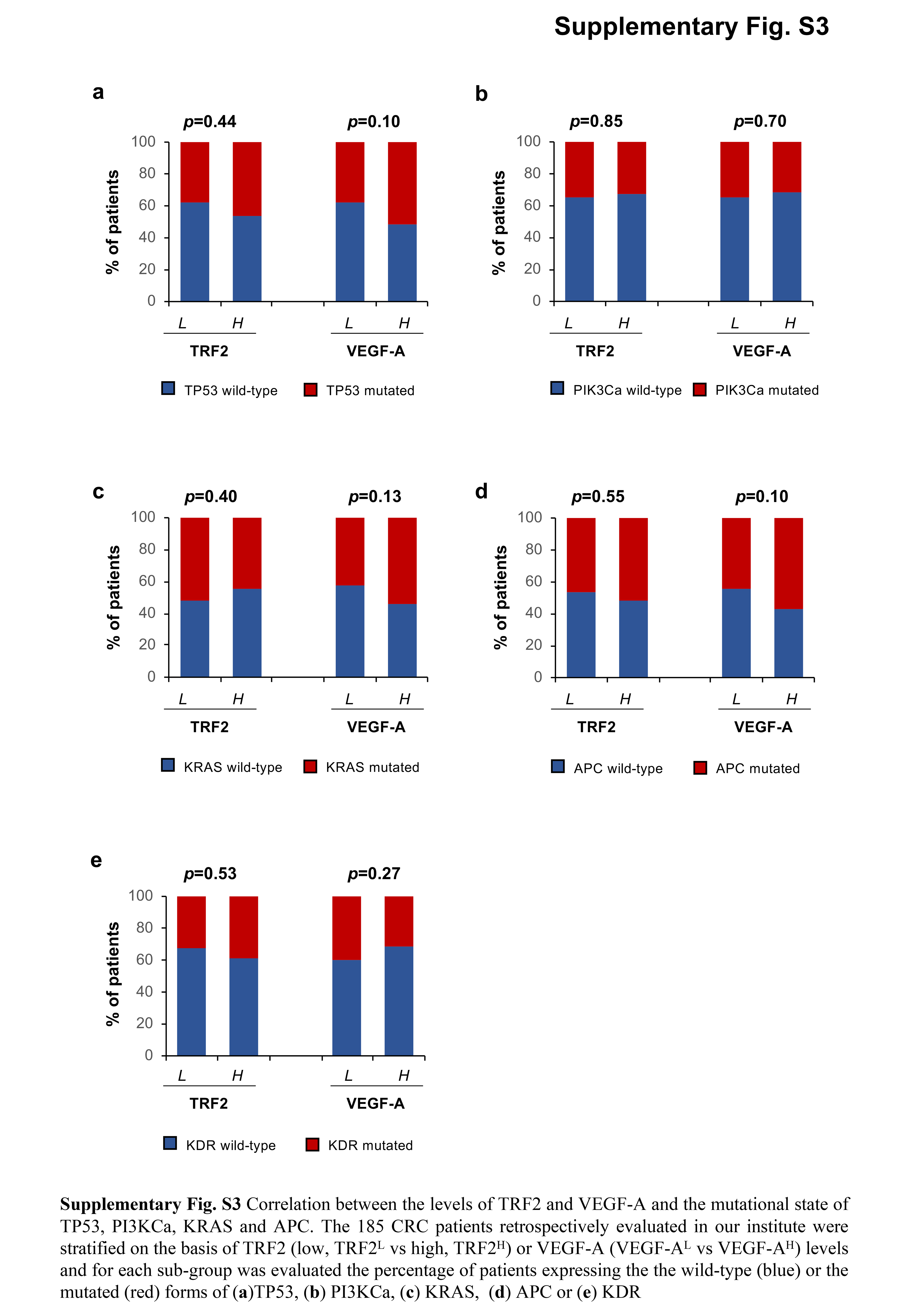

Supplement: Supplementary file 9 — Additional file 9: Supplementary Fig. S3. Correlation between the levels of TRF2 and VEGF-A and the mutational state of TP53, PI3KCa, KRAS and APC [file 13046_2020_1612_MOESM9_ESM.tif]

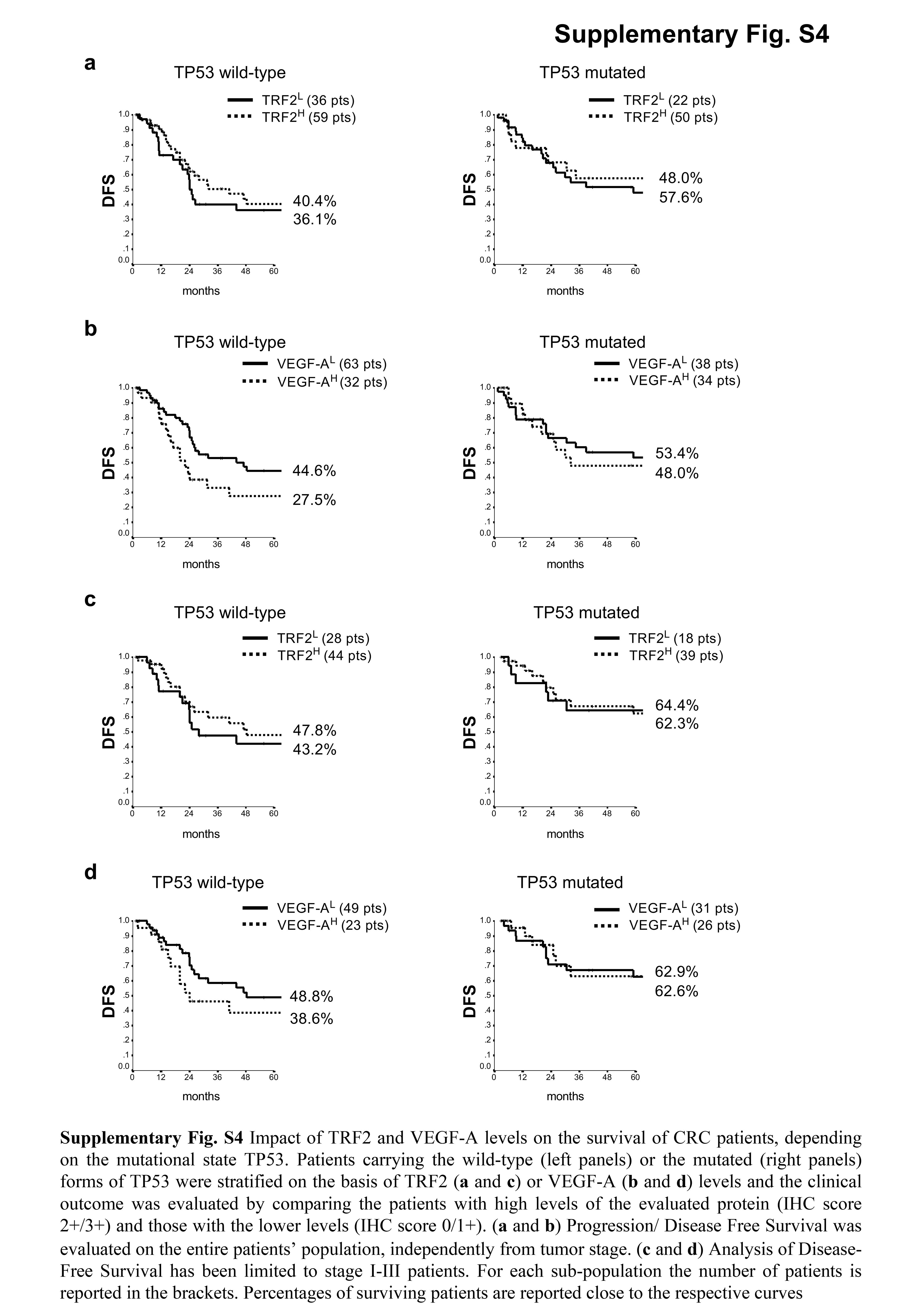

Supplement: Supplementary file 10 — Additional file 10:Supplementary Fig. S4. Impact of TRF2 and VEGF-A levels on the survival of CRC patients, depending on the mutational state of TP53 [file 13046_2020_1612_MOESM10_ESM.tif]

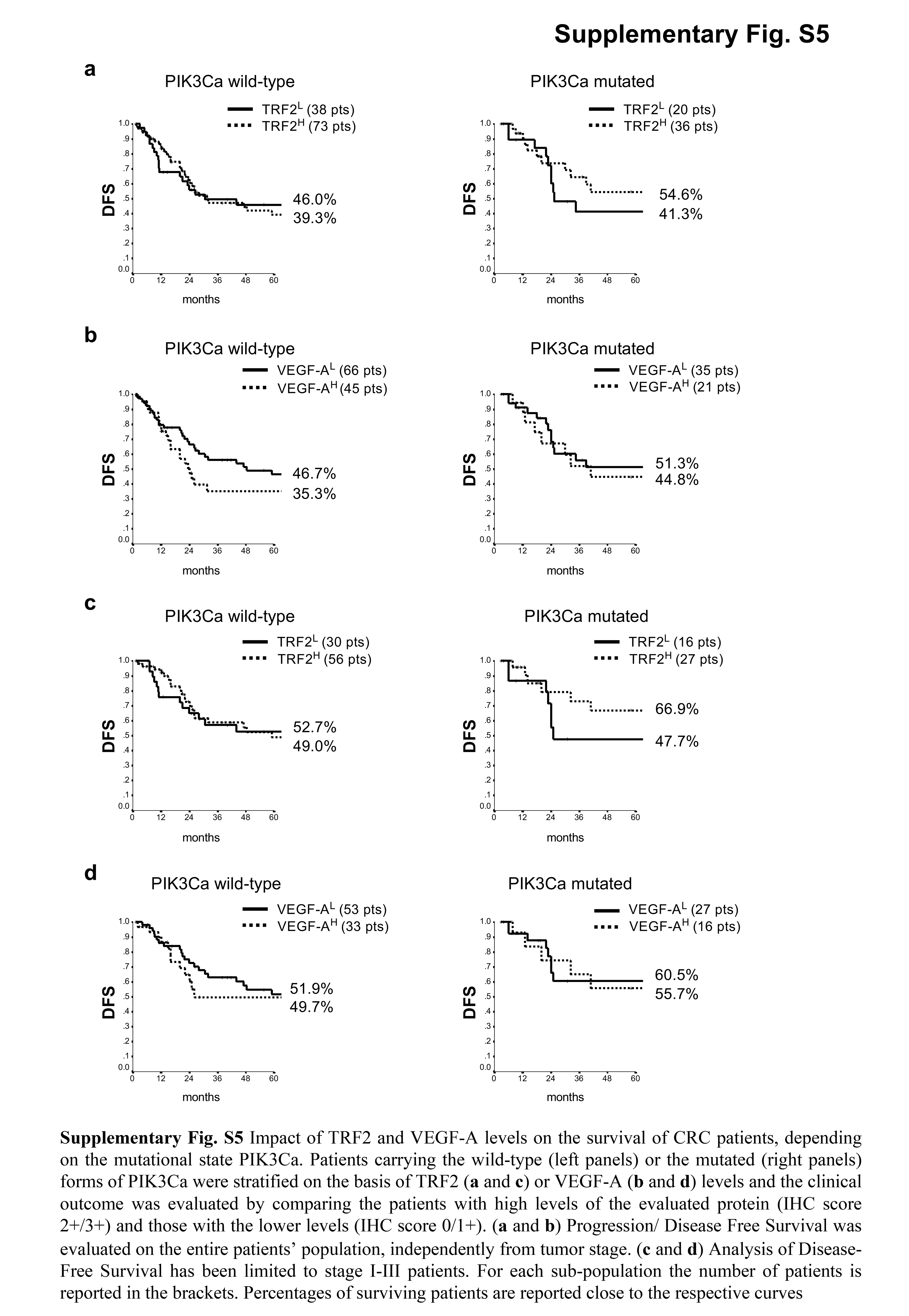

Supplement: Supplementary file 11 — Additional file 11: Supplementary Fig. S5. Impact of TRF2 and VEGF-A levels on the survival of CRC patients, depending on the mutational state of PIK3Ca [file 13046_2020_1612_MOESM11_ESM.tif]

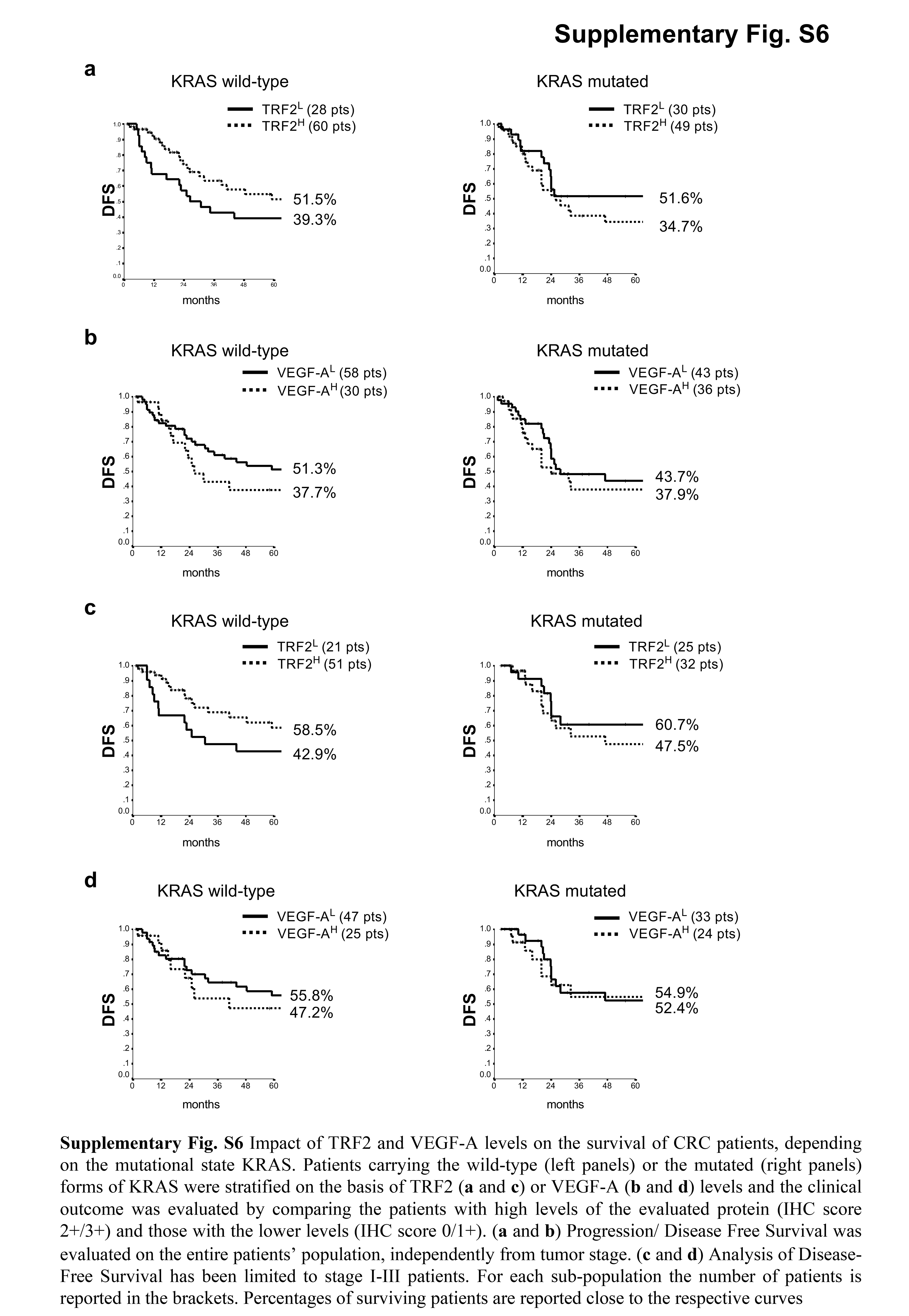

Supplement: Supplementary file 12 — Additional file 12: Supplementary Fig. S6. Impact of TRF2 and VEGF-A levels on the survival of CRC patients, depending on the mutational state of KRAS [file 13046_2020_1612_MOESM12_ESM.tif]

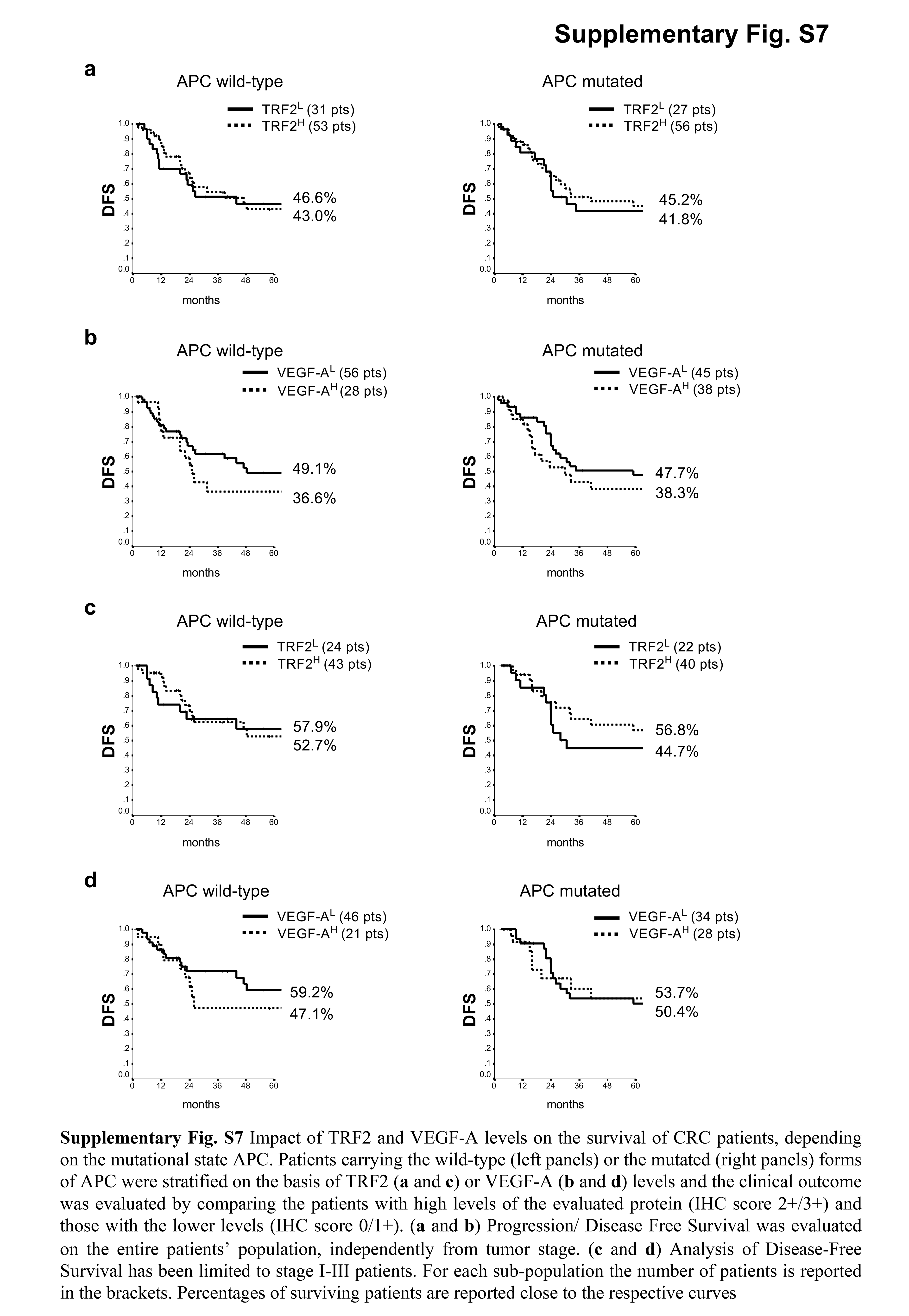

Supplement: Supplementary file 13 — Additional file 13. Supplementary Fig. S7. Impact of TRF2 and VEGF-A levels on the survival of CRC patients, depending on the mutational state of APC [file 13046_2020_1612_MOESM13_ESM.tif]

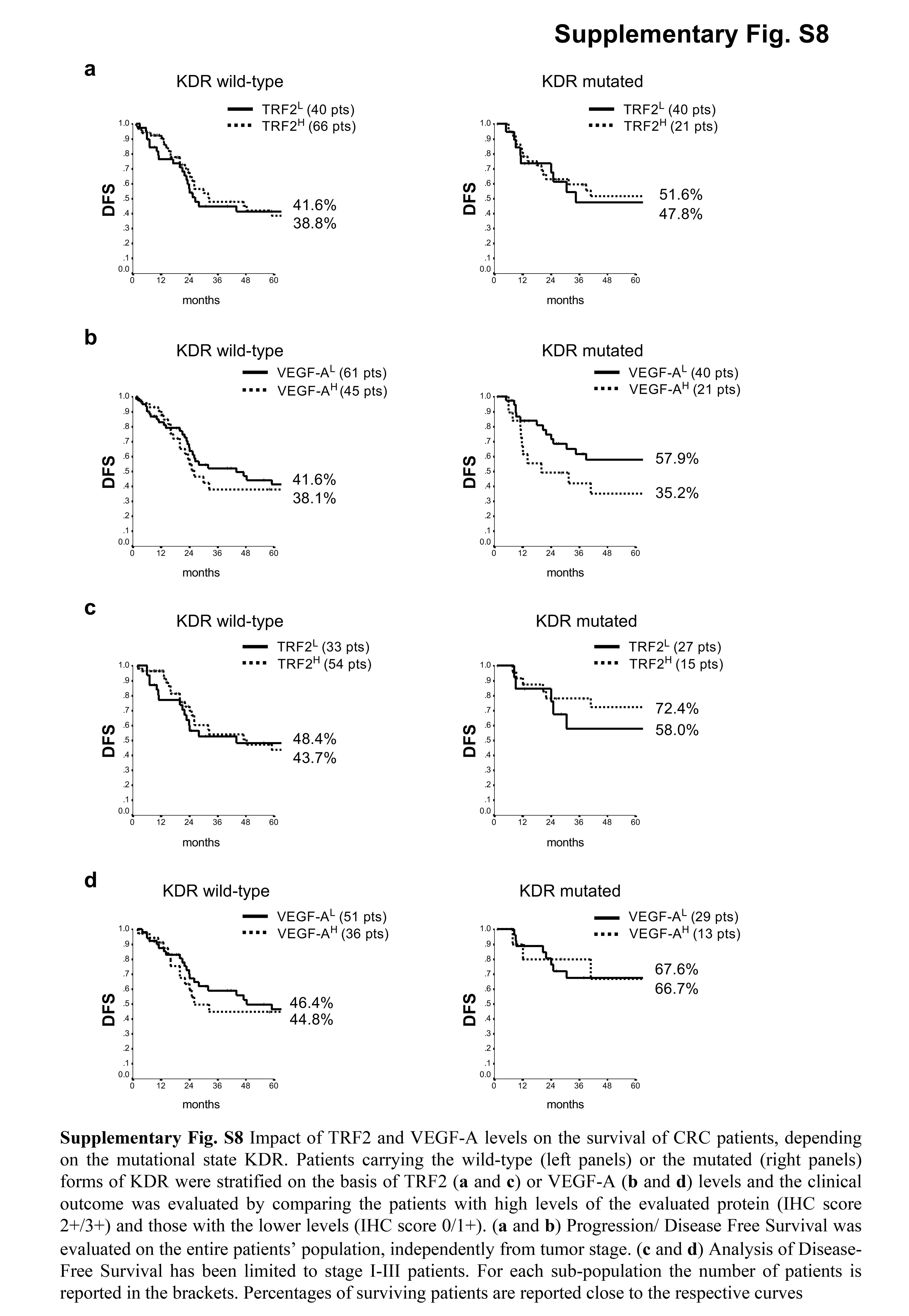

Supplement: Supplementary file 14 — Additional file 14:Supplementary Fig. S8. Impact of TRF2 and VEGF-A levels on the survival of CRC patients, depending on the mutational state of KDR [file 13046_2020_1612_MOESM14_ESM.tif]

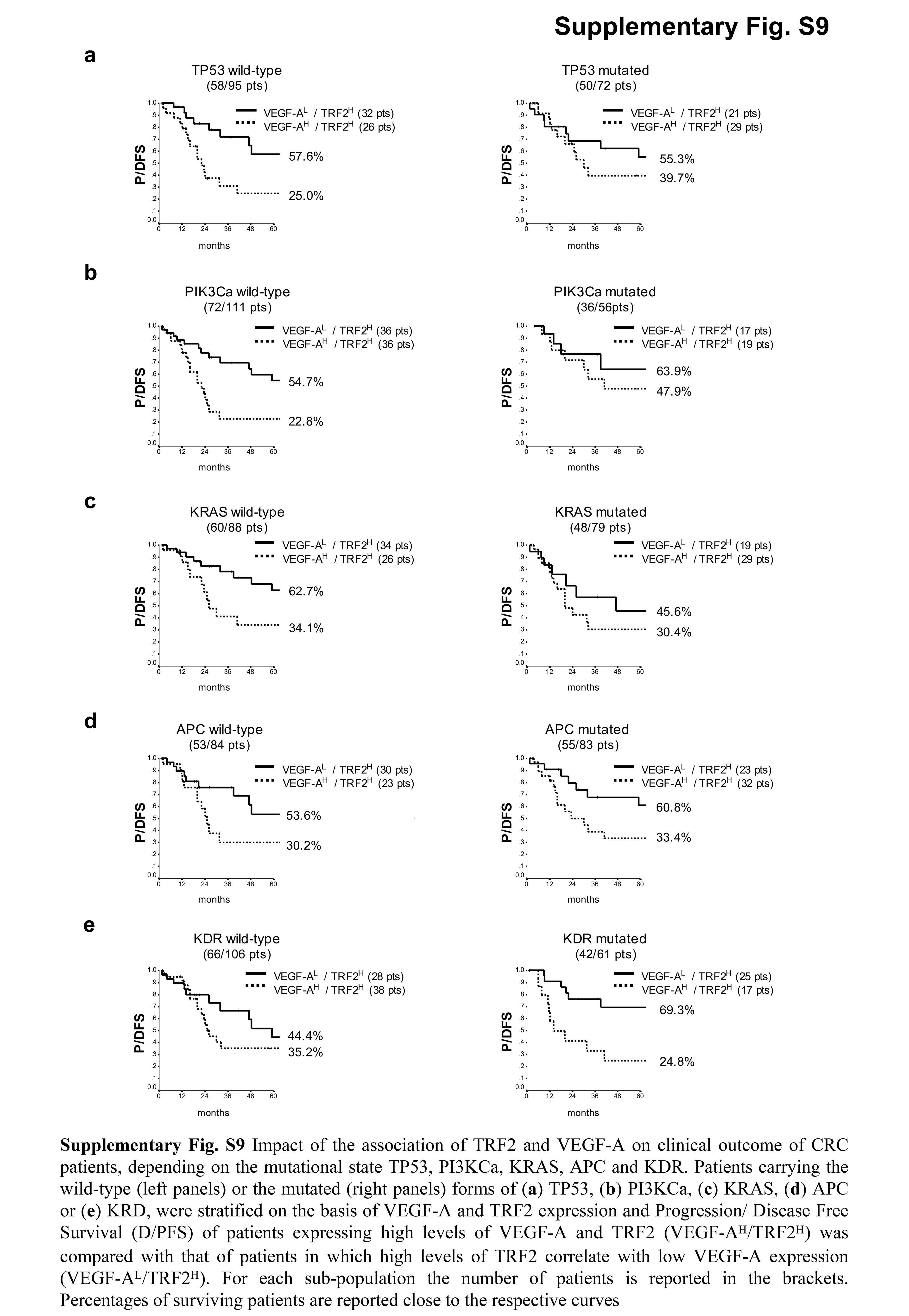

Supplement: Supplementary file 15 — Additional file 15: Supplementary Fig. S9. Imapct of the association of TRF2 and VEGF-A on clinical outcome of CRC patients, depending on the mutational state of TP53, PI3KCa, KRAS, APC and KDR [file 13046_2020_1612_MOESM15_ESM.tif]

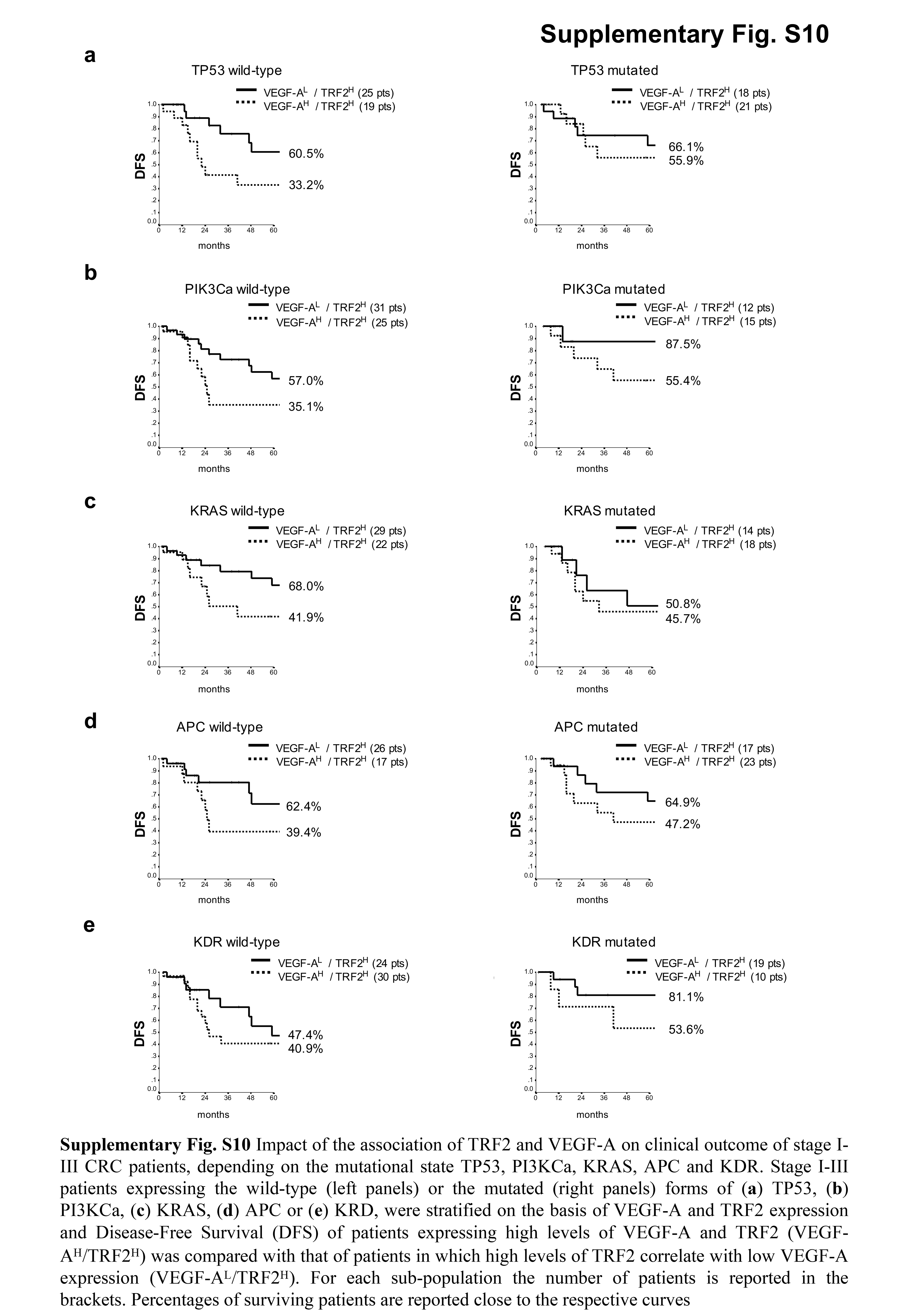

Supplement: Supplementary file 16 — Additional file 16: Supplementary Fig. S10. Impact of the association of TRF2 and VEGF-A on clinical outcome of stage I-III CRC patients, depending on the mutational state of TP53, PI3KCa, KRAS, APC and KDR [file 13046_2020_1612_MOESM16_ESM.tif]

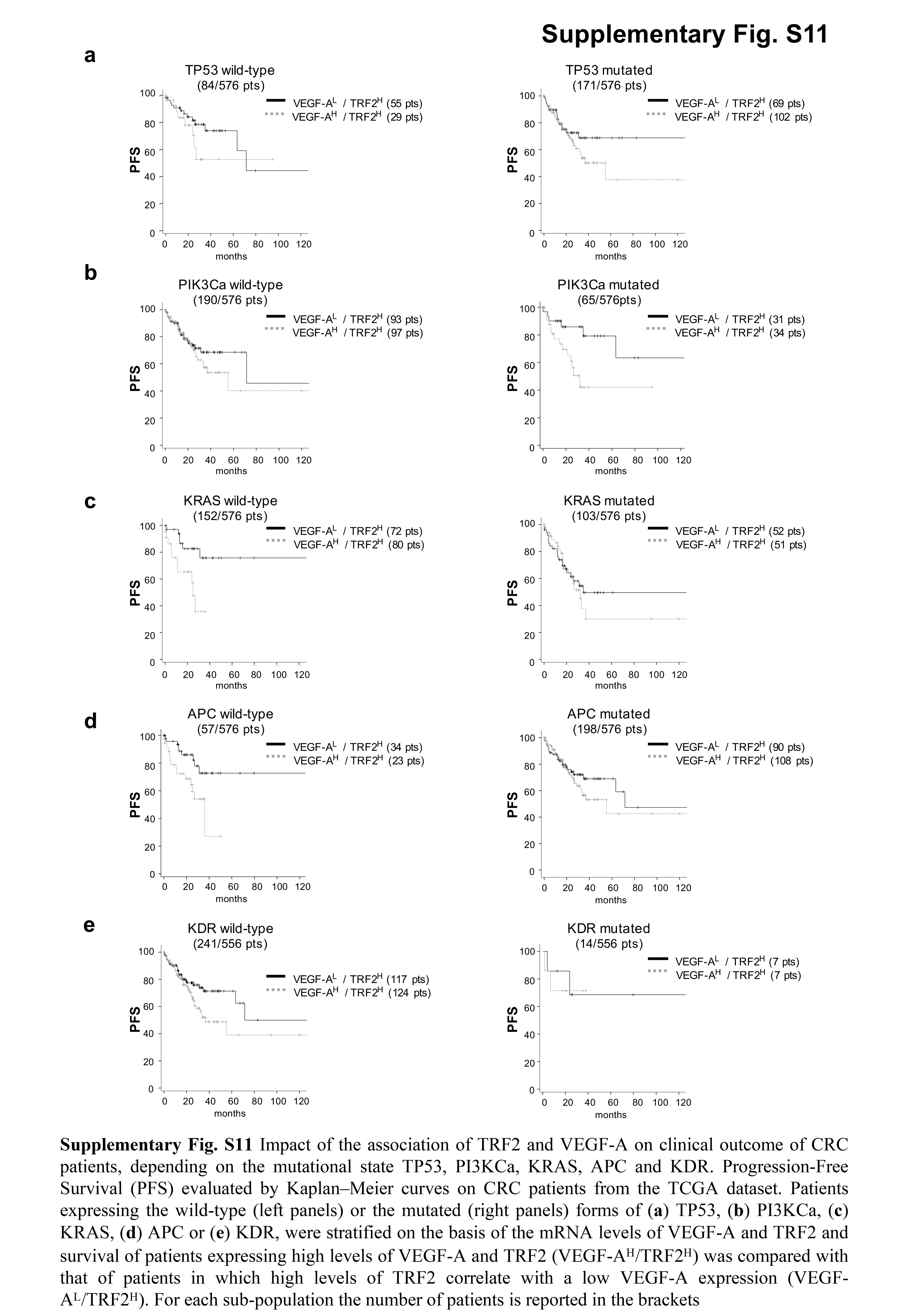

Supplement: Supplementary file 17 — Additional file 17:Supplementary Fig. S11. Impact of the association of TRF2 and VEGF-A on clinical outcome of CRC patients, depending on the mutational state of TP53, PI3KCa, KRAS, APC and KDR [file 13046_2020_1612_MOESM17_ESM.tif]

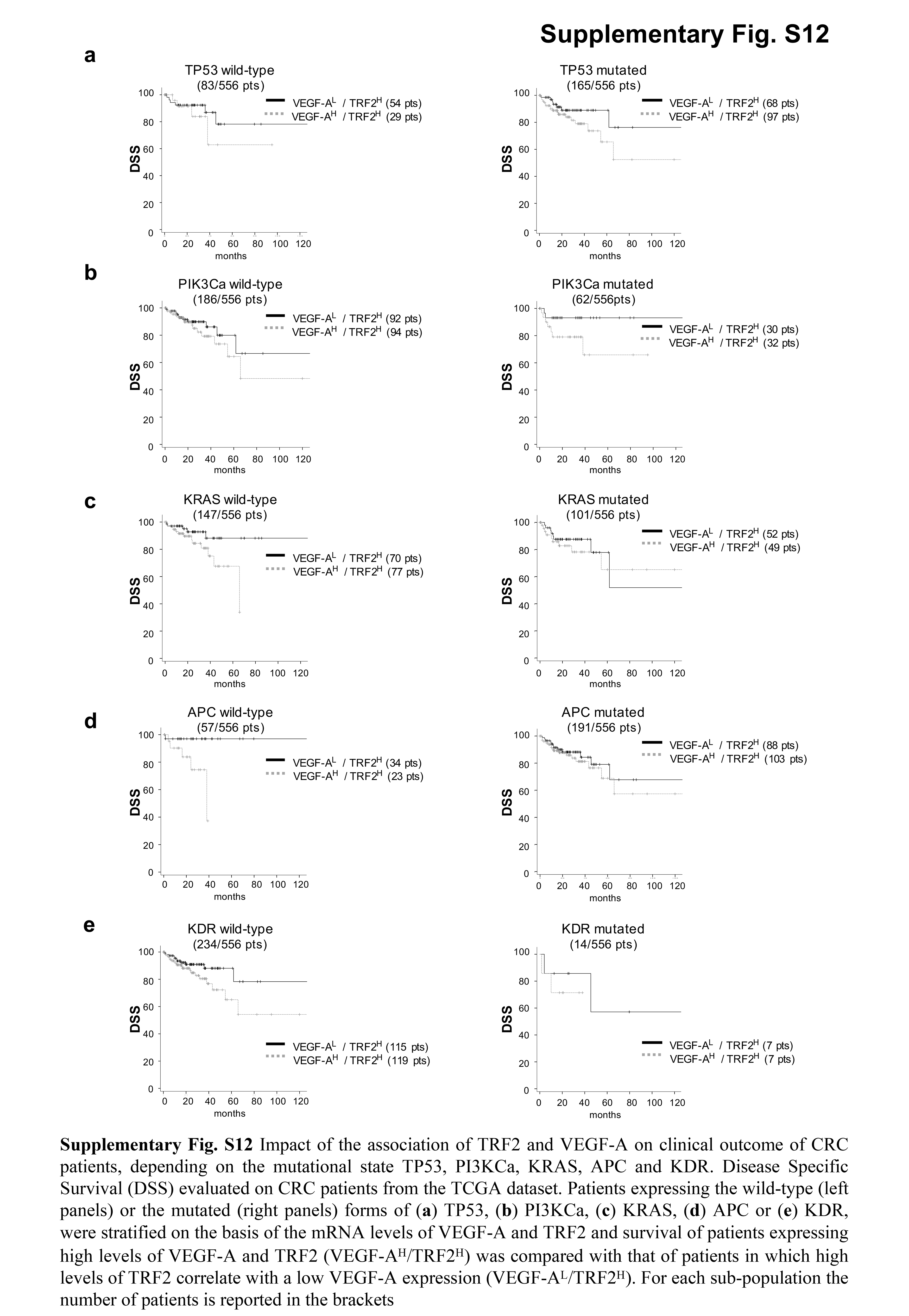

Supplement: Supplementary file 18 — Additional file 18: Supplementary Fig. S12. Impact of the association of TRF2 and VEGF-A on clinical outcome of CRC patients, depending on the mutational state of TP53, PI3KCa, KRAS, APC and KDR [file 13046_2020_1612_MOESM18_ESM.tif]

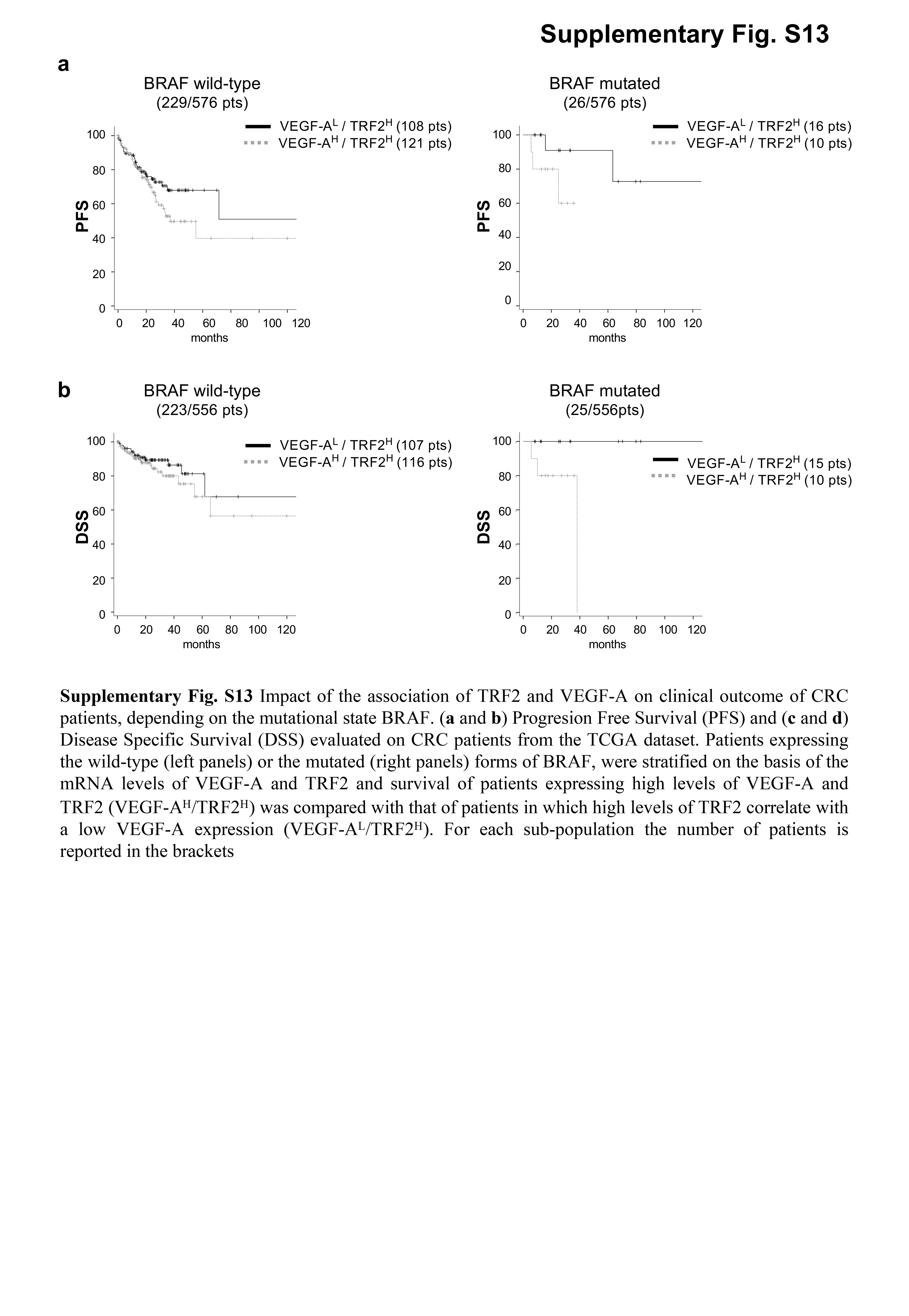

Supplement: Supplementary file 19 — Additional file 19: Supplementary Fig. S13. Impact of the association of TRF2 and VEGF-A on clinical outcome of CRC patients, depending on the mutational state of BRAF [file 13046_2020_1612_MOESM19_ESM.tif]
